# Comparative systematic review and meta-analysis of reactogenicity, immunogenicity and efficacy of vaccines against SARS-CoV-2

**DOI:** 10.1038/s41541-021-00336-1

**Published:** 2021-05-13

**Authors:** Ian McDonald, Sam M. Murray, Catherine J. Reynolds, Daniel M. Altmann, Rosemary J. Boyton

**Affiliations:** 1 Department of Infectious Disease, Faculty of Medicine, Imperial College London, London, UK; 2 Department of Immunology and Inflammation, Faculty of Medicine, Imperial College London, London, UK; 3 Lung Division, Royal Brompton and Harefield Hospitals, London, UK

**Keywords:** Vaccines, Adaptive immunity, Infectious diseases

## Abstract

As SARS-CoV-2 vaccines are deployed worldwide, a comparative evaluation is important to underpin decision-making. We here report a systematic literature review and meta-analysis of Phase I/II/III human trials and non-human primates (NHP) studies, comparing reactogenicity, immunogenicity and efficacy across different vaccine platforms for comparative evaluation (updated to March 22, 2021). Twenty-three NHP and 32 human studies are included. Vaccines result in mostly mild, self-limiting adverse events. Highest spike neutralizing antibody (nAb) responses are identified for the mRNA-1273-SARS-CoV and adjuvanted NVX-CoV2373-SARS-CoV-2 vaccines. ChAdOx-SARS-CoV-2 produces the highest T cell ELISpot responses. Pre-existing nAb against vaccine viral vector are identified following AdH-5-SARS-CoV-2 vaccination, halving immunogenicity. The mRNA vaccines depend on boosting to achieve optimal immunogenicity especially in the elderly. BNT162b2, and mRNA-1273 achieve >94%, rAd26/5 > 91% and ChAdOx-SARS-CoV-2 > 66.7% efficacy. Across different vaccine platforms there are trade-offs between antibody binding, functional nAb titers, T cell frequency, reactogenicity and efficacy. Emergence of variants makes rapid mass rollout of high efficacy vaccines essential to reduce any selective advantage.

## Introduction

Three novel coronaviruses (HCoV) have crossed into humans during the 21st century. Severe acute respiratory syndrome 1 coronavirus (SARS-CoV-1) emerged in China in 2002/3, with 8096 infections and about 10% case fatalities^1^. In 2012, a SARS-like disease emerged in Saudi Arabia, termed Middle East respiratory syndrome coronavirus (MERS-CoV)^2^, sporadic outbreaks leading to 2519 infections and a case fatality rate of 35%^3^. The knowledge accrued in relation to protective immunity and vaccinology of SARS-CoV-1 and MERS-CoV is pertinent to decoding the principles of protection from the highly related SARS-CoV-2.

Various vaccine candidates were developed following the emergence of SARS-CoV-1 and MERS-CoV, with live-attenuated, DNA, and recombinant viral vectors investigated^4–6^. With an animal model in place, advances in vector design and greater knowledge of disease pathogenesis, there was significant pre-clinical and clinical research towards an effective MERS-CoV vaccine^6^. One MERS-CoV vaccine candidate lowered viral shedding in the dromedary camel reservoir^7^. Development of SARS-CoV-1 vaccines began during the outbreak, but subsided as the threat of a major pandemic decreased^8^.

With respect to the SARS-CoV-2 pandemic, there has been unprecedently rapid vaccine development: the Pfizer-BioNTech and Moderna mRNA vaccines and the Astra Zeneca/Oxford ChadOx vaccines are widely rolled-out in several countries as well as some countries utilizing the Chinese Sinovac inactivated SAR-CoV-2 and Russian Sputnik V adenovirus vaccines, and over 300 candidates at different stages of development, utilizing diverse platforms, including protein subunit with adjuvant, non-replicating viral vectors, RNA, virus-like-particles (VLP), DNA, inactivated- and live-attenuated virus^9,10^. An ideal vaccine is one that can be produced at scale and low cost, is safe, easy to distribute and store, induces strong, protective neutralizing antibody and T cell responses, ideally with a single dose, elicits a durable response that does not recapitulate the waning antibody (Ab) titers seen following natural coronavirus infection, and should be equally suitable for very young, old and immunosuppressed individuals^11^. It should also be technically modifiable to accommodate improving efficacy against emerging variants. To achieve these deliverables in the context of the COVID-19 pandemic, several vaccines may be required. Following an initial period awaiting trial results of vaccine candidates, we have entered a period now in which the public, policy-makers and researchers start to consider detailed, comparative questions raised by the extraordinary, real-life challenges of mass vaccination roll-out during an ongoing, global, pandemic. This has focused greater attention on dosing and boost intervals and the quality, quantity and durability of immune responses, as well as details of the adverse event (AE) profile. Past coronavirus vaccine research has highlighted potential safety concerns related to antibody (Ab)-dependant enhancement (ADE) and induction of Th2-associated lung immunopathology following viral challenge^12,13^.

At a time of intense vaccine development across many diverse platforms, there is an incentive to address the need for a comprehensive appraisal benefiting from experience from coronavirus vaccine trials. The investigated approaches encompass different advantages and disadvantages with respect to immunogenicity, efficacy, durability of immunity, safety profile and ease of manufacture, yet there has thus far been relatively little side-by-side evaluation. The aim here was to evaluate pertinent SARS-CoV-1, MERS-CoV and SARS-CoV-2 studies in humans and NHP published up to the March 22, 2021. In the context of the current pandemic this is pertinent to structuring rational, comparative appraisal and selection of the most practical and effective SARS-CoV-2 vaccines.

## Results

**Fig. 1:**
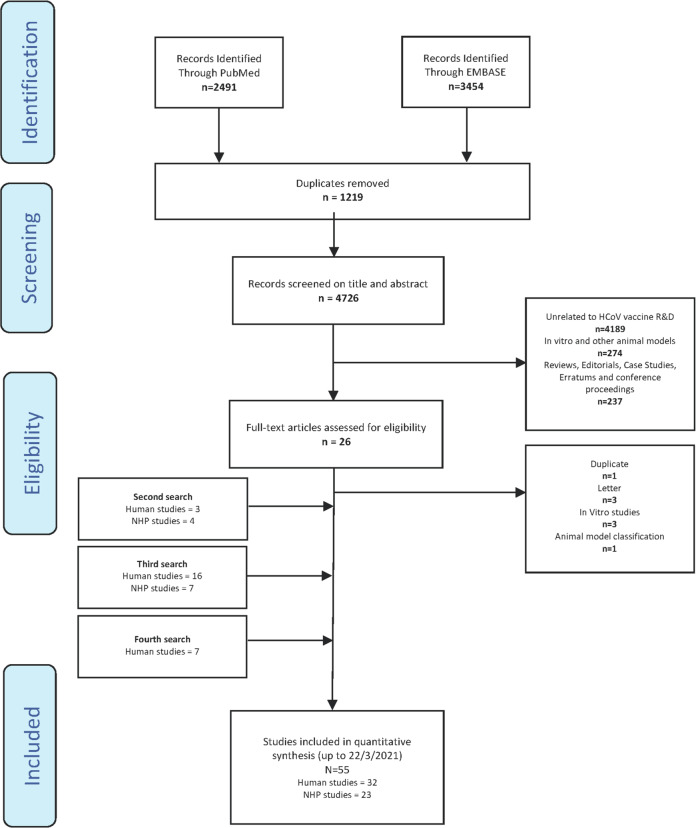
PRISMA diagram. This shows the step-wise process of study selection and pre-determined inclusion and exclusion criteria.

Study selection followed PRISMA guidelines (Fig. 1). A search of PubMed and EMBASE databases used pre-determined search criteria, initially generating 5945 studies (Supplementary Tables 1 and 2). Duplicates, in vitro studies and studies using other animal models were not taken forward. Searches by ‘Title and Abstract’ yielded a total of 55 articles updated to March 22, 2021. Thirty-two human^14–45^ and 23 NHP^46–68^ studies about vaccines against SARS-CoV-1, MERS-CoV and SARS-CoV-2 are reviewed (Supplementary Tables 3 and 4). Individual vaccines are summarized in Table 1 and Supplementary Table 5. The greatest focus of research activity was in China and the USA (Supplementary Fig. 1). The mean age in human trials was 42.7 years. The gender distribution in human vaccine groups was roughly equal. Vaccine group ethnicity was skewed as follows: 72.8% of participants were White, 11.2% Hispanic, 7.2% Black/African American and 3.9% Asian (Supplementary Table 6).

**Table 1:** Summary of human vaccine studies.

Candidate and pathogen	Abbreviation	Vaccine platform	Description	Human	NHP
CanSino, AdH5-nCoV SARS-CoV-2^14,15^	Zhu, AdH5	AdH-5	Replication deficient human adenovirus 5 vectored vaccine expressing optimized full-length spike protein.	☑	
Gamaleya, Gam-COVID-Vac SARS-CoV-2^16,17^	Logunov rAd26/rAd5-Lyo Logunov rAd26/rAd5-Vac Logunov rAd26/rAd5	rAd26/rAd5	Recombinant adenovirus 26 (rAd26) and 5 (rAd5) vectored vaccines expressing full-length spike protein in frozen or lyophilised formulation.	☑	
Janssen, Ad26.COV2.S SARS-CoV-2^18,46^	Sadoff, Ad26.COV2.S	Ad26	Replication deficient adenovirus 26 vectored vaccine expressing stabilised pre-fusion spike protein.	☑	☑
Oxford, ChAdOx1-S-nCoV SARS-CoV-2 and MERS-CoV^19–23,47^	Folegatti, ChAdOx Folegatti, ChAdOx1 MERS Ramasamy, ChAdOx Voysey, ChAdOx	ChAdOx	Replication deficient simian adenovirus vectored vaccine expressing codon-optimized spike protein (SARS-CoV-2) and full-length spike protein (MERS-CoV)	☑	☑
Moderna, mRNA-1273 SARS-CoV-2^24–27,48^	Jackson, mRNA-1273 Anderson, mRNA-1273 Chu, mRNA-1273 Baden, mRNA-1273	mRNA	mRNA vaccine encoding pre-fusion spike protein, encapsulated in a lipid nanoparticle.	☑	☑
Pfizer/BioNTech, BNT162B1/2 SARS-CoV-2^28–31^	Walsh, BNT162b1/2 Mulligan, BNT162b2 Sahin, BNT162b1 Polack, BNT162b2	mRNA	Nucleoside modified mRNA vaccine encoding RBD (BNT162b1) or full-length pre-fusion spike protein (BNT162b2), encapsulated in a lipid nanoparticle.	☑	
CAMS vaccine SARS-CoV-2^32^	Che, inactivated	Inactivated virus (KMS-1 strain)	Formaldehyde and β-propiolactone inactivated vaccine with Aluminium Hydroxide adjuvant.	☑	
Sinopharm, BBIBP-CorV SARS-CoV-2^33,49^	Xia, BBIPB	Inactivated virus (HB02 strain)	β-propiolactone Inactivated vaccine with aluminium hydroxide adjuvant.	☑	☑
Sinovac, CoronaVac/PiCoVac SARS-CoV-2^34,35,50^	Zhang, CoronaVac Wu, CoronaVac	Inactivated virus (CN02 strain)	β-propiolactone Inactivated vaccine with aluminium hydroxide adjuvant.	☑	☑
Bharat, BBV152 (Covaxin) SARS-CoV-2^36,37^	Ella, BBV12	Inactivated virus (NIV-2020–770 strain)	β-propiolactone Inactivated vaccine with TLR7/8 agonist and Alum adjuvant.	☑	
WIBP vaccine SARS-CoV-2^38^	Xia, inactivated	Inactivated virus (WIV04 strain)	β-propiolactone Inactivated vaccine with aluminium hydroxide adjuvant.	☑	
Novavax, NVX-CoV2373 SARS-CoV-2^39,51^	Keech, NVX-CoV2373	Recombinant protein	Recombinant nanoparticle vaccine expressing full-length spike protein with or without the Matrix M1 Adjuvant.	☑	☑
Inovio, INO-4800 SARS-CoV-2^40^	Tebas, INO-4800	DNA plasmid	DNA plasmid vaccine expressing optimised, synthetic full-length spike protein gene	☑	
Clover/GSK, SCB-2019 SARS-CoV-2^41^	Richmond, SCB-2019	Recombinant protein subunit	Stabilised trimeric spike protein vaccine with AS03 or CpG 1018/Alum Adjuvant.	☑	
MVA-MERS-S MERS-CoV^42^	Koch, MERS MVA	rMVA	Modified Vaccina Ankara (Poxvirus) expressing full-length spike protein	☑	
VRC-SRSDNA015–00-VP SARS-CoV-1^43^	Martin, SARS DNA	DNA plasmid	DNA plasmid encoding codon-optimized spike protein gene	☑	
Sinovac-Inactivated SARS-CoV-1^44^	Lin, SARS inactivated	Inactivated virus (Sino3 strain)	β-propiolactone inactivated vaccine with aluminium hydroxide adjuvant.	☑	
GLS-5300 MERS-CoV^45^	Modjarrad, GLS-5300 MERS	DNA plasmid	pGX9101 plasmid expressing the gene for the optimized, full length, microconsensus MERS-CoV spike protein	☑	

There were many different vaccine platforms used, the most frequent being viral vector platforms, followed by nucleic acid (mRNA/DNA) and inactivated virus platforms. Excluding the inactivated vaccine platforms, the predominant antigen insert employed was a variant of, or the full length, spike protein—the defined nAb target. The vast majority of vaccine schedules use a 2-dose prime/boost protocol, with the interval between doses ranging from 14 to 84 days and boost at day 21 being the most commonly reported protocol (Table 1 and Supplementary Tables 3–5).

Antibody binding studies were largely carried out by ELISA-IgG in NHP and human vaccine studies. One NHP study used ELISA-IgA to evaluate mucosal immunity^59^. Antigens used for IgG detection in humans trials were spike GP, spike RBD and trimeric spike GP. T cell analysis was primarily by IFNγ ELISpot or by intracellular cytokine staining (ICS), generally using overlapping peptide pools for spike antigen. We have not attempted a cross-comparison between these two readouts to arrive at a universal comparison of spike-specific responder cell frequency since differential strategies for CD4/CD8 gating in ICS impose a confounder.

Of the five published human studies evaluating vaccine efficacy, the Polack^31^, BNT162b2 (Pfizer-BioNTech) and Baden^27^, mRNA-1273 (Moderna vaccine) vaccines showed the highest efficacy at greater than 94.6% followed by the Logunov^17^, rAd26/rAd5 vaccine at 91.6% efficacy. The Voysey^21,22^, ChAdOx (Astra Zeneca/Oxford Vaccine) demonstrated efficacy of 70.4% and 66.7% in two pooled efficacy studies (Supplementary Table 7). For efficacy and safety analysis of vaccine candidates tested in NHP studies, histopathology, biochemical analysis, clinical and radiological evaluations were most often used, along with RT-PCR measure of viral load (Supplementary Tables 4 and 8).

### Adverse events

NHP safety analysis was described in seven studies (Supplementary Table 8). There were few notable changes in biochemical, hematological or clinical signs in the majority of NHP vaccine groups. Four studies evaluated ADE risk^49,50,54,58^, suggested to be a risk factor in HCoV vaccine safety, and none of the studies found evidence of ADE. No significant Th2 pathology was found in any NHP analysis.

**Fig. 2:**
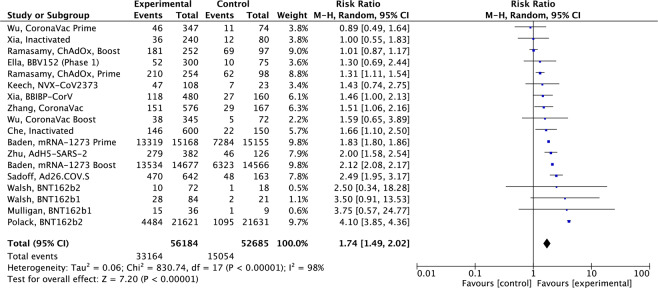
Forest plot analysis of the overall incidence of solicited adverse events (AE) in 15 human studies. A risk ratio of >1 is indicative of a higher incidence of AE in experimental compared to control groups. A Mantel–Haenszel variance random effects model (CI) was employed to estimate effects between experimental and control groups correcting for confounding variables. *I*^2^ statistic, 95% CI and point estimates are displayed for each individual study.

Safety analysis of local and systemic AE were recorded across all 32 human studies. Figure 2 shows a comparative Forest plot analysis of the incidence of AE across published human studies looking at the relative risk in vaccine compared to control groups. Overall, there is a 1.7-fold increased risk of any AE in the vaccinated compared to control groups. There was a 4.1-fold increased risk in Polack^31^, BNT162b2 mRNA vaccine and a 1.8-fold increased risk in the Baden^27^, mRNA-1273 vaccine (Fig. 2). The vast majority of reported AE were in the mild local and systemic category.

**Fig. 3:**
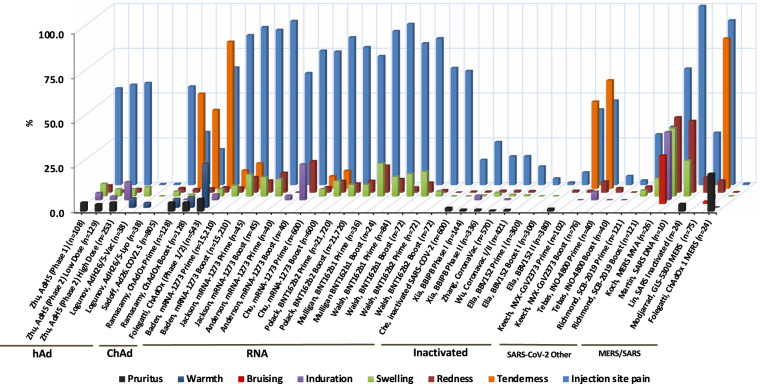
Interstudy analysis of local adverse events reported in vaccine group (excluding control group) of coronavirus vaccine studies. Symptoms are represented by different colors. The percent (%) of vaccines reporting any grade of AE is shown. First author and vaccine platform are recorded on the *X* axis. hAd human adenoviral, ChAd chimpanzee adenoviral.

In terms of mild or moderate local AE, the most commonly reported local AE was injection site pain, followed by redness and swelling (Fig. 3 and Supplementary Table 9). Martin DNA-SARS-CoV-1^43^, Modjarrad DNA-MERS-CoV^45^, Baden/Jackson/Anderson/Chu mRNA-1273-SARS-CoV-2^24–27^, Walsh/Mulligan/Pollack BNT162b1/2-SARS-CoV-2^28,29,31^, Logunov rAd26/rAd5-SARS-COV-2^16,17^ and Folegatti/Ramasamy, SARS-CoV-2 ChAdOx^19,20^ and Keech NVX-CoV2373^39^ vaccines all reported a high percentage of subjects with injection site pain. Koch MERS-CoV^42^ and Martin DNA-SARS-CoV-1^43^ reported the highest incidence of local redness and swelling.

**Fig. 4:**
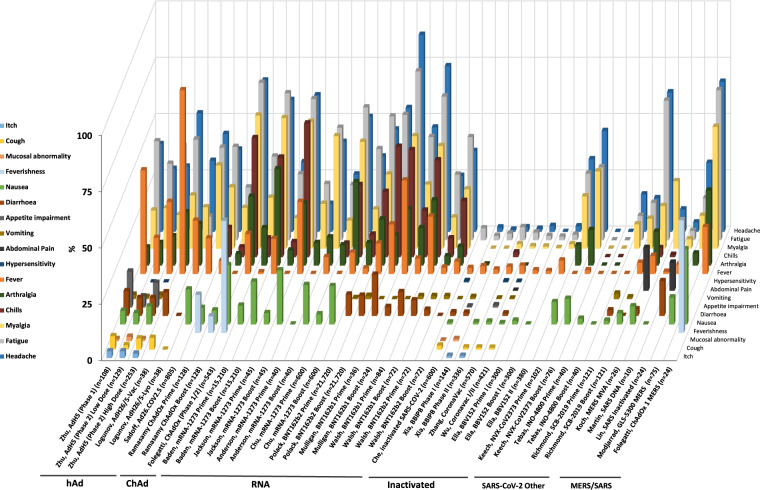
Interstudy analysis of systematic adverse events reported in vaccine group (excluding control group) of coronavirus vaccine studies. Symptoms are represented by different colors. The percent (%) of vaccines reporting any grade of symptomatic AE is shown. First author and vaccine platform are recorded on the *X* axis. hAd human adenoviral, ChAd chimpanzee adenoviral.

In terms of mild or moderate systemic AEs, the most commonly reported were headache, fever, myalgia, chills and fatigue (Fig. 4 and Supplementary Table 10). They were reported for most vaccine platforms, including BNT162b1/2-SARS-CoV-2^28,29,31^, mRNA-1273-SARS-CoV-2^24–27^, SARS-CoV-2 ChAdOx^19,20^, NVX-CoV2373^39^ and rAd26/rAd5-SARS-COV-2 (Sputnik V)^16,17^ vaccines. There is increased frequency of mild/moderate systemic AE events with increased dose and at the boost of a prime boost schedule seen in the RNA vaccines Baden/Jackson/Anderson mRNA-1273-SARS-CoV-2^24,25,27^, and Walsh/Mulligan/Pollack BNT162b1/2-SARS-CoV-2^28,29,31^, and Keech, NVX1273-Cov^39^ vaccines with increased incidence of headache, myalgia, arthralgia and fatigue after boosting. Analysis of individual local and systemic AE is available in Figs. 3 and 4 and Supplementary Tables 9 and 10.

Several of the clinical trials included in this systematic review recorded grade three AE related to vaccine or placebo (Supplementary Fig. 2). Baden^27^, mRNA-1273 showed the highest percentage of grade 3 AE (11%) following boost. Zhu^14,15^, AdH5, Keech^39^, NVX-CoV2373, Sadoff^18^, Ad26.CoV2.S, Mulligan/Walsh^28,29^ BNT162b1/2 and Folegatti^23^, ChAdOx all reported grade 3 AE in more than 3.5% of participants.

The occurrence of reported solicited and unsolicited serious AE following SARS-CoV-2 vaccination in the published studies to date was low with similar reporting in vaccine and control groups at 0.6% in each (Baden^27^, mRNA-1273); 0.6% vaccine and 0.5% control (Pollack^31^, BNT162b2); 0.7% vaccine and 0.8% control (Voysey^21^, ChAdOx) and 0.3% vaccine and 0.4% control (Logunov^17^, rAd26/rAd5) (Supplementary Table 11).

Autonomic nervous imbalance, nausea, vomiting, rheumatoid arthritis, dyspnea, swollen face and peripheral edema were reported in the vaccine group of Baden, mRNA-1273^27^. Transverse myelitis and fever were recorded in the experimental arm of Voysey, ChAdOx-SARS-CoV-2^21^. Shoulder injury related to vaccination, right axillary lymphadenopathy, paroxysmal ventricular arrhythmia and right leg paresthesia were recorded in the Polack, BNT162b2-SARS-CoV-2^31^.

### Antibody responses

**Fig. 5:**
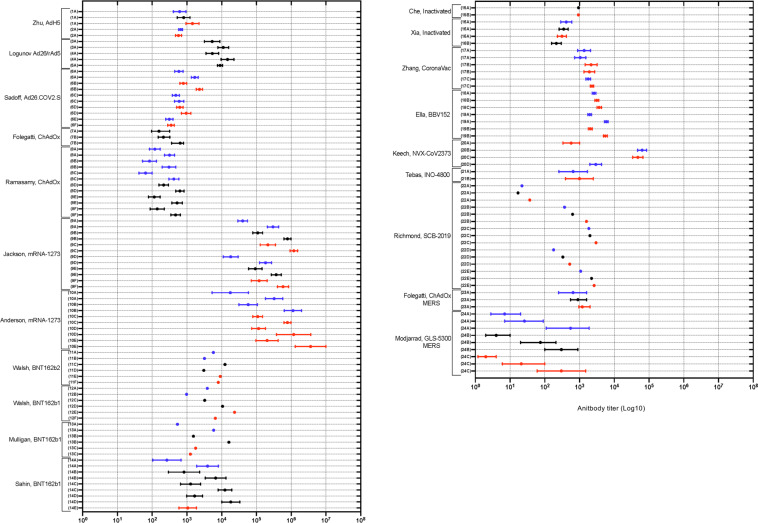
Forest plot of log-transformed ELISA-IgG antibody titre following HCoV vaccination in human studies. Blue = low; black = intermediate; red = high dose of vaccine indicated. Antibody titre recorded circa 28 days post injection. Log transformed scale used. Vp viral particles, RBD receptor binding domain, GMT geometric mean titre, CI confidence interval, S2-P SARS-CoV-2 GP with transmembrane anchor and intact S1-S2 cleavage site, S1 S1 spike glycoprotein domain, GMET geometric mean endpoint titre, EU ELISA unit, IQR interquartile range. (**1A**) Zhu, AdH-5, Phase 1: low dose—5 × 10^10^ vp, intermediate dose—1 × 10^11^ vp, high dose —1.5 × 10^11^ vp; RBD; expressed as GMT (95% CI); (**2A**) Zhu, AdH-5, Phase 2: low dose—1 × 10^11^ vp, high dose—5 × 10^10^ vp; RBD; expressed as GMT (95% CI); (**3A**) Logunov, rAdH26/rAd5-Lyo: lyophilised formulation. Unspecified dose—10^11^ vp; Prime-Boost; RBD; expressed as GMT (95% CI); (**4A**) Logunov, rAdH26/rAd5-Vac: frozen formulation. Unspecified dose—10^11^ vp; Prime-Boost; RBD; expressed as GMT (95% CI); (**5A**) Logunov, rAdH26/rAd5-Vac Phase 3 trial: unspecified dose—10^11^ vp; post boost in a Prime-Boost; RBD; expressed as GMT (95% CI); (**6A-F**) Sadoff, Ad26.COV2.S: low dose—5 × 10^10^ vp, high dose—1 × 10^11^ vp; (**A**) Prime-Boost (low), (**B**) Prime-Boost (high), (**C**) Prime-Placebo (low), (**D**) Prime-Placebo (high), (**E**) ≥ 65yo, Prime (high), (**F**) ≥ 65yo, Prime (low); stabilized pre-fusion spike protein; expressed as EU (GMC, 95% CI); (**7A**, **B**) Folegatti, ChAdOx SARS-CoV-2: unspecified dose—5 × 10^10^ vp; (**A**) Prime only, (**B**) Prime-Boost; trimeric spike protein; expressed as EU (IQR); (**8A**–**F**) Ramasamy, ChAdOx: low dose—2.2 × 10^10^ vp, unspecified dose— 3.5–6.5 × 10^10^ vp; (**A**) Prime-Boost, 18–55 y/o (low), (**B**) Prime-Boost, 56–69 y/o (low), (**C**) Prime-Boost, ≥70 y/o (low), (**D**) Prime-Boost, 18–55 y/o (unspecified), (**E**) Prime-Boost, 56–69 y/o (unspecified), (**F**) Prime-Boost, ≥70 y/o (unspecified); Spike protein; expressed as GMT (95% CI); (**9A-F**) Jackson,mRNA-1273: low dose—25 µg, intermediate dose—100 µg, high dose—250 µg; (**A**) Prime-Boost (low) S2-P, (**B**) Prime-Boost (intermediate) S2-P, (**C**) Prime-Boost (high) S2-P, (**D**) Prime-Boost (low) RBD, (**E**) Prime-Boost (intermediate) RBD, (**F**) Prime-Boost (High) RBD; Expressed as GMT (95% CI); (**10A-E**) Anderson, mRNA-1273: low dose—25 µg, high dose—100 µg; **A**) Boost, 56–70 y/o (low), (**B**) Boost, ≥70 y/o (low), (**C**) Boost, 18–55 y/o (high), (**D**) Boost, 56–70 y/o (high), (**E**) Boost, ≥70 y/o (high); S-2P; expressed as GMT (95% CI); (**11A-F**) Walsh, BNT162b2: low dose—10 µg, intermediate dose—20 µg, high dose—30 µg; (**A**) Boost, 18–55 y/o (low), (**B**) Boost, 65–85 y/o (low), (**C**) Boost, 18–55 y/o (intermediate), (**D**) Boost, 65–85 y/o (intermediate), (**E**) Boost, 18–55 y/o (high), (**F**) Boost, 65–85 y/o (high); S1; expressed as GMT; (**12A-F**) Walsh, BNT162b1: low dose—10 µg, intermediate dose—20 µg, high dose—30 µg; (**A**) Boost, 18–55 y/o (low), **B**) Boost, 65–85 y/o (low), (**C**) Boost, 18–55 y/o (intermediate), (**D**) Boost, 65–85 y/o (intermediate), (**E**) Boost, 18–55 y/o (high), (**F**) Boost, 65–85 y/o (High); S1; expressed as GMT; (**13A-C**) Mulligan, BNT162b1: (**A**) Prime-Boost (10 µg dose), (**B**) Prime-Boost (30 µg dose), (**C**) Prime-Boost (100 µg dose); RBD; expressed as GMT; (**14A-E**) Sahin, BNT162b1: (**A**) Prime-Boost (1 µg dose), (**B**) Prime-Boost (10 µg dose), (**C**) Prime-Boost (30 µg), (**D**) Prime-Boost (50 µg), (**E**) Prime (60 µg); RBD; expressed as GMT (95% CI); (**15A**, **B**) Che, inactivated: intermediate dose—100 EU, high dose—150 EU; Spike protein; expressed as GMET (95% CI); (**16A**) Xia, inactivated Phase 1: low dose—2.5 µg, intermediate dose—5 µg, high dose—10 µg, 3rd boost of Prime-Boost; inactivated SARS-CoV-2; expressed as GMT (95% CI); (**16B**) Xia, inactivated Phase 2: intermediate dose—5 µg 3rd Boost of Prime-Boost; inactivated SARS-CoV-2; expressed as GMT (95% CI); (**17A**, **B**) Zhang, CoronaVac, Phase 1: (**A**) Prime-Boost (low dose—3 µg), (**B**) Prime-Boost (high dose—6 µg); RBD; expressed as GMT (95% CI); (**17C**) Zhang, CoronaVac, Phase 2: low dose—3 µg, high dose—6 µg, 2nd boost in a Prime-Boost; RBD; expressed as GMT (95% CI); (**18A**–**C**) Ella, BBV152 Phase 1: low dose—3 µg, high dose—6 µg; (**A**) post prime (low + Algel-IMDG), (**B**) Post prime (high + Algel-IMDG), (**C**) post prime (high + Algel); RBD; expressed as GMT (95% CI); (**19A**, **B**) Ella, BBV152 Phase 2: (**A**) Prime-Boost (low dose 3 µg + Algel-IMDG), (**B**) Prime-Boost (high dose 6 µg + Algel-IMDG); RBD; expressed as GMT (95% CI); (**20A**–**D**) Keech, NVX-CoV2373: low dose—5 µg, high dose—25 µg; (**A**) Prime-Boost (high), (**B**) Prime-Boost (Low + adjuvant), (**C**) Prime-Boost (high + adjuvant), (**D**) Prime-Boost (high + placebo); Spike protein; expressed as EU (GMT, 95% CI); (**21A**, **B**) Tebas, INO-4800: low dose—1.0 mg, high dose—2.0 mg; Boost in a Prime-Boost; (**A**) low dose, (**B**) high dose; Spike Protein (S1/2); expressed as reciprocal binding titre (median, IQR); (**22A**–**E**) Richmond, SCB-2019: low dose—3 µg, intermediate dose—9 µg, high dose—30 µg; (**A**) Boost, 18–54 y/o (low, intermediate, high, non-adjuvanted). (**B**) Boost, 18–54 y/o (low, intermediate, high + CpG/Alum), (**C**) Boost, 18–54 y/o (low, intermediate, high + AS03), (**D**) Boost, 55–75 y/o (low, intermediate, high, CpG/Alum), (**E**) Boost, 55–75 y/o (low, intermediate, high + AS03); SCB-2019 binding antibody; expressed as GMT (95% CI); (**23A**) Folegatti, ChAdOx-MERS-CoV: low dose—5 × 10^9^ vp, intermediate dose—2.5 × 10^10^ vp, high dose—5 × 10^10^ vp; Spike; expressed as EU (95% CI); (**24A–C**) Modjarrad, GLS-500-MERS-CoV: low dose—0.67 mg, intermediate dose—2 mg, high dose—6 mg, (**A**) Prime-Boost (low), (**B**) Prime-Boost (intermediate), (**C**) Prime-Boost (high); Spike protein; expressed as GMET (95% CI).

Interstudy analysis of ELISA-IgG GMT was conducted across published human studies^14–20,23–25,28–30,32,34,36–41,45^. A number of conclusions can be drawn regarding overall titer and the dose-dependent and boost dependent nature of ELISA-IgG Ab responses (Fig. 5).

The vaccine achieving the highest Ab titer was the Jackson, Anderson mRNA-1273-SARS-CoV-2^24,25^ vaccine. It exhibited dose and boosting dependent increases in Ab titer. The high-dose Keech NVX-CoV2373^39^ vaccine elicited similar Ab titers to the unboosted mRNA-1273-SARS-CoV-2 vaccine. The next highest antibody binding titers were seen in the Sahin, Walsh BNT162b1-SARS-CoV-2^28,30^ and Logunov^16^, rAd26/rAd5-SARS-COV-2 followed by Ella, BBV152^36,37^, Sadoff, Ad26^18^ and Folegatti/Ramasamy, SARS-CoV-2 ChAdOx^19,20^. Several vaccines display boost-dependant increases in Ab titer including Logunov AdH26-SARS-CoV-2^16^; Ramasamy, Folegatti ChAdOx-SARS-CoV-2^19,20^; and Sahin, Walsh BNT162b1-SARS-CoV-2^28,30^. Modjarrad DNA-MERS-CoV^45^ elicited similar Ab responses to the Zhu, AdH-5-SARS-CoV-2^14,15^ and Folegatti, ChAdOx-MERS-CoV^23^ vaccines, and exhibited dose-dependent increase in responses in the middle and high dose vaccine group. The inactivated virus vaccines included in this review achieved similar Ab responses with minimal boost or dose-dependent increases in titers^32,38,44^.

Comparing human and NHP GMT ELISA-IgG responses, human studies reported mean Ab titers similar to ELISA-IgG responses seen in NHP studies (Supplementary Fig. 3A).

**Fig. 6:**
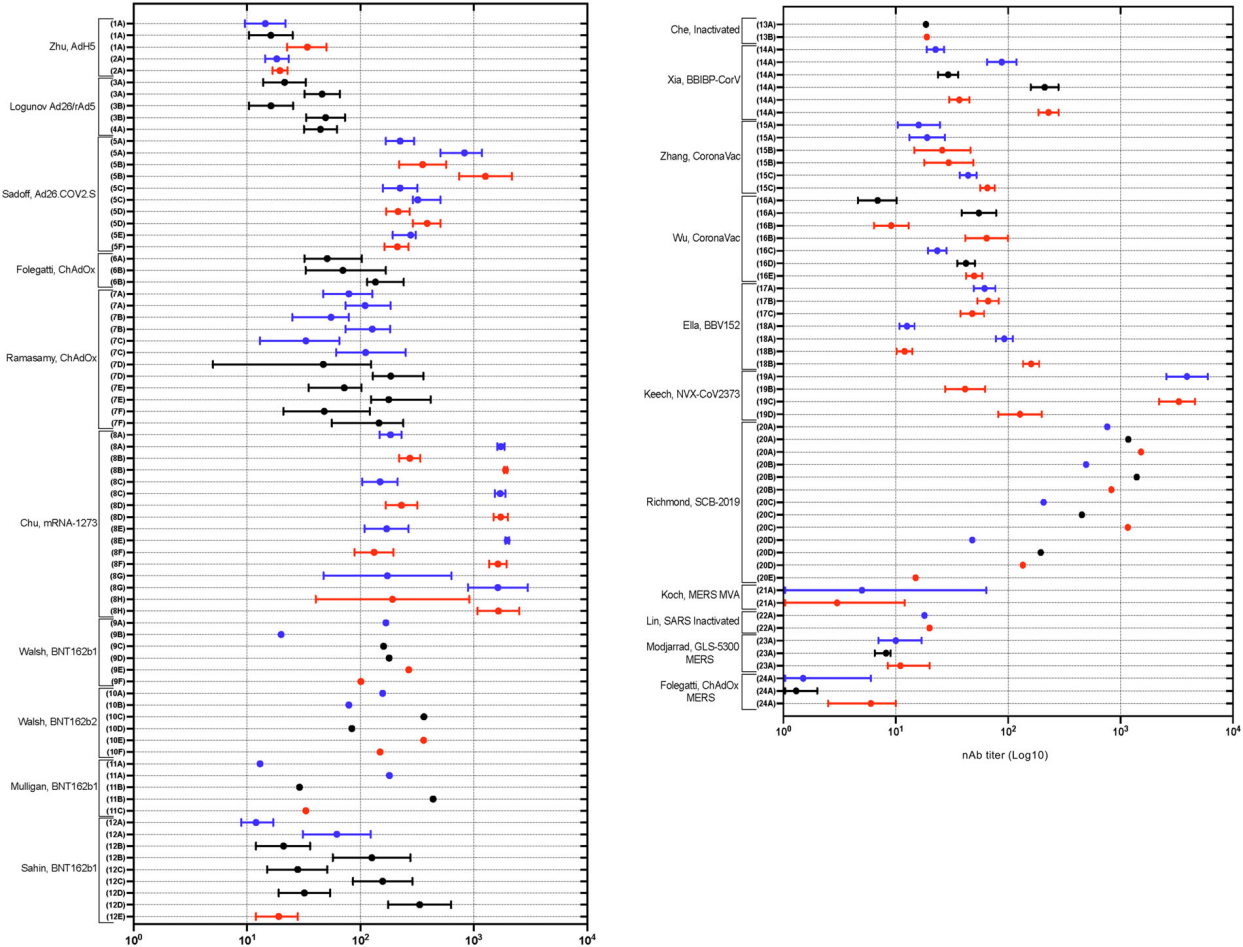
Forest plot of nAb antibody titres following HCoV vaccination in human studies. Blue = low; black = intermediate; red = high dose of vaccine indicated. Neutralizing antibody titre recorded circa 28 days post injection. Log transformed scale used. Vp viral particles, GMT geometric mean titre, CI confidence interval, PFU, plaque-forming units, GMET geometric mean endpoint titre, IQR interquartile range, CCID cell culture infectious dose, TCID tissue culture infectious dose, IC inhibitory concentration, SU SARS-CoV-1 units. (**1A**) Zhu, AdH-5, Phase 1: low dose—5 × 10^10^ vp, intermediate dose—1 × 10^11^ vp, high dose: 1.5 × 10^11^ vp; live SARS-CoV-2 neutralization assay; expressed as GMT (95% CI); (**2A**) Zhu, AdH-5, Phase 2: low dose—1 × 10^11^ vp, high dose—5 × 10^10^ vp; live SARS-CoV-2 neutralization assay; expressed as GMT (95% CI); (**3A**) Logunov, rAdH26/rAd5-Lyo: lyophilised formulation. Unspecified dose—10^11^ vp; Prime-Boost; microneutralization assay, 100TCID_50_, expressed as GMT (95% CI); (**3B**) Logunov, rAdH26/rAd5-Vac: frozen formulation. Unspecified dose—10^11^ vp; Prime-Boost; microneutralization assay, 100TCID_50_, expressed as GMT (95% CI); (**4A**) Logunov, rAdH26/rAd5-Vac Phase 3 trial: unspecified dose—10^11^ vp. Post boost in a Prime-Boost; microneutralization assay TCID50; expressed as GMT (95% CI); (**5A**–**F**) Sadoff, Ad26: low dose—5 × 10^10^ vp, high dose—1 × 10^11^ vp; (**A**) Prime-Boost (low), (**B**) Prime-Boost (high), (**C**) Prime-Placebo (low), (**D**) Prime-Placebo (high), (**E**) ≥ 65yo, Prime (high), (**F**) ≥ 65yo, Prime (low); live SARS-CoV-2 neutralization assay (IC_50_); expressed as GMT (95% CI); (**6A**, **B**) Folegatti, ChAdOx SARS-CoV-2: unspecified dose—5 × 10^10^ vp; (**A**) Prime only, (**B**) Prime-Boost; microneutralization assay (MNA_50_); expressed as GMT (IQR); (**7A–F**) Ramasamy, ChAdOx: low dose—2.2 × 10^10^ vp, unspecified dose—3.5–6.5 × 10^10^ vp; (**A**) Prime-Boost, 18–55 y/o (low), (**B**) Prime-Boost, 56–69 y/o (low), (**C**) Prime-Boost, ≥70 y/o (low), (**D**) Prime-Boost, 18–55 y/o (unspecified), (**E**) Prime-Boost, 56–69 y/o (unspecified), (**F**) Prime-Boost, ≥70 y/o (unspecified); microneutralization assay (MNA_80_; normalised IC_50_ values); (**8A**–**H**) Chu, mRNA-1273: low dose—50 µg, high dose—100 µg. (**A**) Prime-Boost, 18–55 y/o (low), (**B**) Prime-Boost, 18–55 y/o (high), (**C**) Prime-Boost, 55–65 y/o (low), (**D**) Prime-Boost, 55–65 y/o (high), (**E**) Prime-Boost, 65–75 y/o (low), (**F**) Prime-Boost, 65–75 y/o (high), (**G**) Prime-Boost, ≥75 y/o (low), (**H**) Prime-Boost, ≥75 y/o (High); live virus microneutralization assay (MN_50_). Expressed as GMT (95% CI); (**9A**–**F**) Walsh, BNT162b1: low dose—10 µg, intermediate dose—20 µg, high dose—30 µg; (**A**) Boost, 18–55 y/o (low), (**B**) Boost, 65–85 y/o (low), (**C**) Boost, 18–55 y/o (intermediate), (**D**) Boost, 65–85 y/o (intermediate), (**E**) Boost, 18–55 y/o (high), (**F**) Boost, 65–85 y/o (high); fluorescent-based neutralization assay, 50% neutralization titres expressed as GMT (95% CI); (**10A**–**F**) Walsh, BNT162b2: low dose—10 µg, intermediate dose—20 µg, high dose—30 µg; (**A**) Boost, 18–55 y/o (low), (**B**) Boost, 65–85 y/o (low), (**C**) Boost, 18–55 y/o (intermediate), (**D**) Boost, 65–85 y/o (intermediate), (**E**) Boost, 18–55 y/o (high), (**F**) Boost, 65–85 y/o (high); fluorescent-based neutralization assay, 50% neutralization titres expressed as GMT (95% CI); (**11A–C**) Mulligan, BNT162b1: (**A**) Prime-Boost (10 µg dose), (**B**) Prime-Boost (30 µg dose), (**C**) Prime (100 µg dose); fluorescent-based neutralization assay, 50% neutralization titres expressed as GMT, (95% CI); (**12A**–**E**) Sahin, BNT162b1: (**A**) Prime-Boost (1 µg dose), (**B**) Prime-Boost (10 µg dose), (**C**) Prime-Boost (30 µg), (**D**) Prime-Boost (50 µg), (**E**) Prime (60 µg); fluorescent-based neutralization assay, 50% neutralization titres expressed as GMT, VNT_50_ (95% CI); (**13A**, **B**) Che, inactivated: intermediate dose—100 EU, high dose—150 EU; live SARS-CoV-2 neutralisation assay (CCID_50_); expressed as GMT (95% CI); (**14A**) Xia, BBIBP-CorV: low dose—2 µg, intermediate dose—4 µg, high dose— 8 µg, Prime-Boost; live SARS-CoV-2 neutralization assay; expressed as GMT (95% CI); (**15A-B**) Zhang, CoronaVac, Phase 1: (**A**) Prime-Boost (low dose—3 µg), (**B**) Prime-Boost (high dose—6 µg); live SARS-CoV-2 neutralization assay; expressed as GMT (95% CI); (**15C**) Zhang, CoronaVac, Phase 2: low dose—3 µg, high dose—6 µg; 2nd boost in a Prime-Boost; live SARS-CoV-2 neutralization assay; expressed as GMT (95% CI); (**16A**, **B**) Wu, CoronaVac: Phase 1: intermediate dose—3 µg, high dose—6 µg; (**A**) Prime-Boost, ≥60 y/o (intermediate), (**B**) Prime-Boost, ≥60 y/o (high); live SARS-CoV-2 neutralization assay; expressed as GMT (95% CI); (**16C**–**E**) Wu, CoronaVac: Phase 2: low dose—1.5 µg, intermediate dose—3 µg, high dose—6 µg; (**C**) Boost, ≥60 y/o (low), (**C**) Boost, ≥60 y/o (intermediate), (**C**) Boost, ≥60 y/o (high); live SARS-CoV-2 neutralization assay; expressed as GMT (95% CI); (**17A**–**C**) Ella, BBV152 Phase 1: low dose—3 µg, high dose—6 µg; (**A**) post prime (low + Algel-IMDG), (**B**) post prime (high + Algel-IMDG), (**C**) post prime (high + Algel); microneutralization assay (MNT_50_). Expressed as GMT (95% CI); (**18A**, **B**) Ella, BBV152 Phase 2: (**A**) Prime-Boost (low dose 3 µg + Algel-IMDG), (**B**) Prime-Boost (high dose 6 µg + Algel-IMDG); microneutralization assay (MNT_50_). Expressed as GMT (95% CI); (**19A**–**D**) Keech, NVX-CoV2373: low dose—5 µg, high dose—25 µg; (**A**) Prime-Boost (5 µg +adjuvant), (**B**) Prime-Boost (25 µg no adjuvant), (**C**) Prime-Boost (25 µg + adjuvant), (**D**) Prime-Boost (25 µg + Placebo); microneutralization assay (IC_99_); expressed as GMT (95% CI); (**20A**) Richmond, SCB-2019**:** low dose—3 µg, intermediate dose—9 µg, high dose—30 µg; (**A**) Boost, 18–54 y/o (low, intermediate, high, AS03). (**B**) Boost, 55–75 y/o (low, intermediate, high + AS03), (**C**) Boost, 18–54 y/o (Low, intermediate, High + CpG/Alum), (**D**) Boost, 55–75 y/o (low, intermediate, High, CpG/Alum). (**E**) Boost, 18–54 y/o (high non-adjuvanted); SCB-2019 binding antibody. Expressed as GMT (95% CI); (**21A**) Koch, MVA-MERS-CoV: low dose—1 × 10^7^ PFU, high dose—1 × 10^8^ PFU Boost in a Prime-Boost; live MERS-CoV neutralization assay; expressed as the GMT (95% CI); (**22A**) Lin, Inactivated-SARS-CoV-1: low dose—16 SU, high dose—32 SU; live virus neutralization assay (CCID_50_); expressed as GMT (95% CI); (**23A**) Modjarrad, GLS-500-MERS-CoV: low dose—0.67 mg, intermediate dose—2 mg, high dose—6 mg; 2nd Boost in a Prime-Boost; live MERS-CoV neutralization assay (TCID_50_); expressed as GMET (95% CI); (**24A**) Folegatti, ChAdOx-MERS-CoV: low dose—5 × 10^9^ vp, intermediate dose—2.5 × 10^10^ vp, high dose—5 × 10^10^ vp; live MERS-CoV neutralization assay (TCID_50_); expressed as GMT (95% CI).

**Fig. 7:**
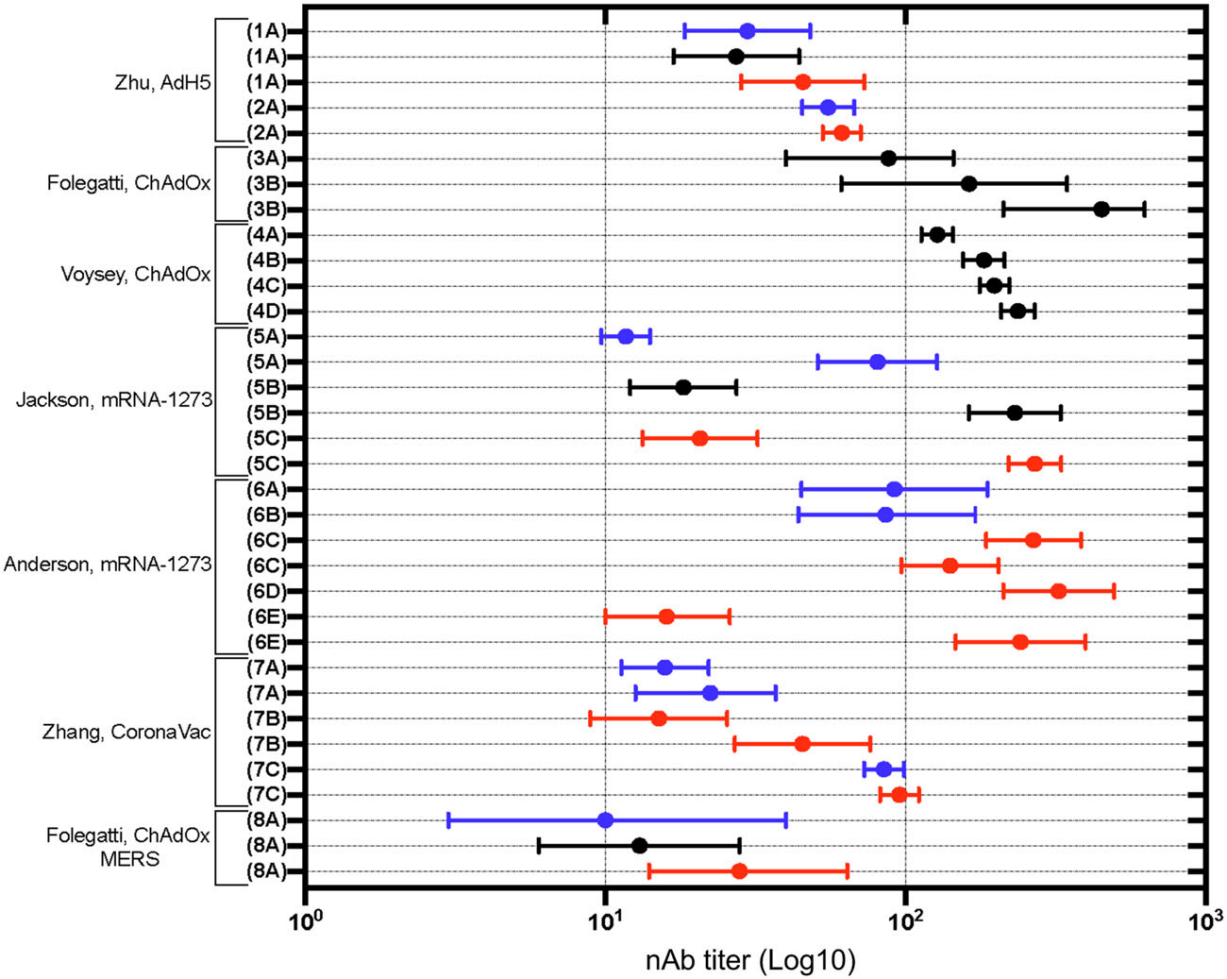
Forest plot of pseudotyped nAb titres following HCoV vaccination in human studies. Blue = low; black = intermediate; red = high dose of vaccine indicated. Neutralizing antibody titre recorded circa 28 days post injection. Log transformed scale used. Vp Viral particles, GMT geometric mean titre, CI confidence interval, PFU plaque-forming units, GMET geometric mean endpoint titre, IQR interquartile range, CCID cell culture infectious dose, TCID tissue culture infectious dose, IC inhibitory concentration, SU SARS-CoV-1 units, ID infectious dose, IC inhibitory concentration. (**1A**) Zhu, AdH-5 Phase 1: low dose—5 × 10^10^ vp, intermediate dose—1 × 10^11^ vp, high dose—1.5 × 10^11^ vp; VSV pseudovirus system expressing spike protein; expressed as GMT (95% CI); (**2A**) Zhu, AdH-5 Phase 2: low dose—1 × 10^11^ vp, high dose—5 × 10^10^ vp; VSV pseudovirus system expressing spike protein; expressed as GMT (95% CI); (**3A**, **B**) Folegatti, ChAdOx SARS-CoV-2: unspecified dose—5 × 10^10^ vp; (**A**) Prime, (**B**) Prime-Boost; lentivirus pseudovirus system (IC_50_) expressed as median titre (IQR); (**4A**–**D**) Voysey, ChAdOx: unspecified dose—5 × 10^10^vp; (**A**) Prime-Boost, <6 week interval, (**B**) Prime-Boost, 5–8 week interval, (**C**) Prime-Boost, 9–11 week interval, (**D**) Prime-Boost, ≥12 week interval; lentivirus pseudovirus system (IC_50_) expressed as GMT (95% CI); (**5A**–**C**) Jackson, mRNA-1273: low dose—25 µg, intermediate dose—100 µg, high dose—250 µg; (**A**) Prime-Boost (low), (**B**) Prime-Boost (intermediate), (**C**) Prime-Boost (high); lentivirus pseudovirus system expressing spike protein (ID_50_); expressed as GMT (95% CI); (**6A**–**E**) Anderson, mRNA-1273: low dose—25 µg, high dose—100 µg; (**A**) Boost, 56–70 y/o (low), (**B**) Boost, ≥70 y/o (low), (**C**) Prime-Boost, 18–55 y/o (high), (**D**) Boost, 56–70 y/o (high), (**E**) Prime-Boost, ≥70 y/o (high); lentivirus pseudovirus system expressing spike protein (ID_50_); expressed as GMT (95% CI); (**7A**, **B**) Zhang, CoronaVac, Phase 1: low dose—3 µg, high dose—6 µg; (**A**) Prime-Boost (low), (**B**) Prime-Boost (high); pseudovirus neutralisation assay; expressed as GMT (95% CI); (**7C**) Zhang, CoronaVac, Phase 2: low dose—3 µg, high dose—6 µg, 2nd boost in a Prime-Boost; pseudovirus neutralisation assay; expressed as GMT (95% CI); (**8A**) Folegatti, ChAdOx-MERS-CoV: low dose—5 × 10^9^ vp, intermediate dose—2.5 × 10^10^ vp, high dose—5 × 10^10^ vp; lentivirus pseudovirus system expressing spike protein (IC_50_); expressed as GMT (95% CI).

Next we looked at human vaccine trial data for nAb responses (Fig. 6). The adjuvanted Keech, NVX-CoV2373-SARS-CoV-2^39^ and Chu, mRNA-1273^26^ Prime-Boost vaccine groups achieved the highest nAb responses followed by the Sadoff, Ad26-SARS-CoV2^18^ vaccine then Folegatti/Ramasamy, SARS-CoV-2 ChAdOx^19,20^ and Sahin, Walsh BNT162b1-SARS-CoV-2^28,30^. Both are dependent on the boost to achieve high nAb titers. At the vaccination dose of 30 µg, the 56–85 yo cohort of the Walsh, BNT162B1/2^28^ study achieved lower nAb titers than the 15–55 yo cohorts. A similar reduction in nAb titers was seen with increasing age across the cohorts (18–55 yo, 56–70 yo and >70 yo) in Ramasamy, SARS-CoV-2 ChAdOx^20^. This emphasizes the importance of the boosting dose to achieve optimal nAb titers particularly in older age groups. The importance of a boost dose to achieve optimal nAb titers is also seen in the Jackson/Anderson mRNA-1273^24,25^ and Folegatti, SARS-CoV-2 ChAdOx^19^ studies using a pseudotyped virus nAb assay (Fig. 7). Data from the >70 yo age group in the Anderson, mRNA-1273 study^25^ (Fig. 7) is noteworthy as boosting is needed to achieve a measurable titer. In Chu, mRNA-1273^26^ there was no reduction in nAb response with increasing age.

Studies looking at human and NHP live and pseudovirus nAb responses to vaccination report responses of a similar order of magnitude (Supplementary Fig. 3B, C).

A number of vaccines display convergence between ELISA, pseudotype virus neutralization and live virus nAb titers. In the Anderson mRNA-1273-SARS-CoV vaccine groups^25^ there were significant dose-dependent increases in pseudotype virus nAb titer, similar to the ELISA-IgG responses (Figs. 5, 6, and 7). A similar relationship exists between the live nAb and ELISA-IgG responses in the Sahin, BNT162b1^30^, Logunov, AdH26/5-Vac/Lyo^16^ and Ramasamy, ChAdOx SARS-CoV-2^20^ vaccines.

Studies have reported pre-existing Ab against the vaccine viral vector itself. Supplementary Table 12 details pre-existing titers: the Zhu, AdH-5-SARS-CoV-2^14,15^ vectored vaccines showed high levels of pre-existing nAb with an average of 52.2% (range: 44–58%) of participants having pre-existing nAb (>1:200, titer) to the AdH-5 virus vector. Pre-existing Ab roughly halved the Ab response to the vaccine. About 9.2% (range: 5–13%) of participants in the Logunov, AdH26/5-Vac/Lyo^16^ trial had pre-existing anti-vector nAb. However, no significant correlation was found between titers of anti-vector nAb and titers of RBD specific IgG. Only one percent of participants in the Folegatti, ChAdOx-SARS-CoV-2^19^ had pre-existing anti-vector nAb of >1:200 although 19% did have nAb. In the larger Ramasamy, ChAdOx-SARS-CoV-2 trial^20^, there were no pre-existing nAbs, however the anti-ChAdOx nAb titer increased following prime vaccination, but did not after boost vaccination (Supplementary Table 12).

### T cell responses

**Fig. 8:**
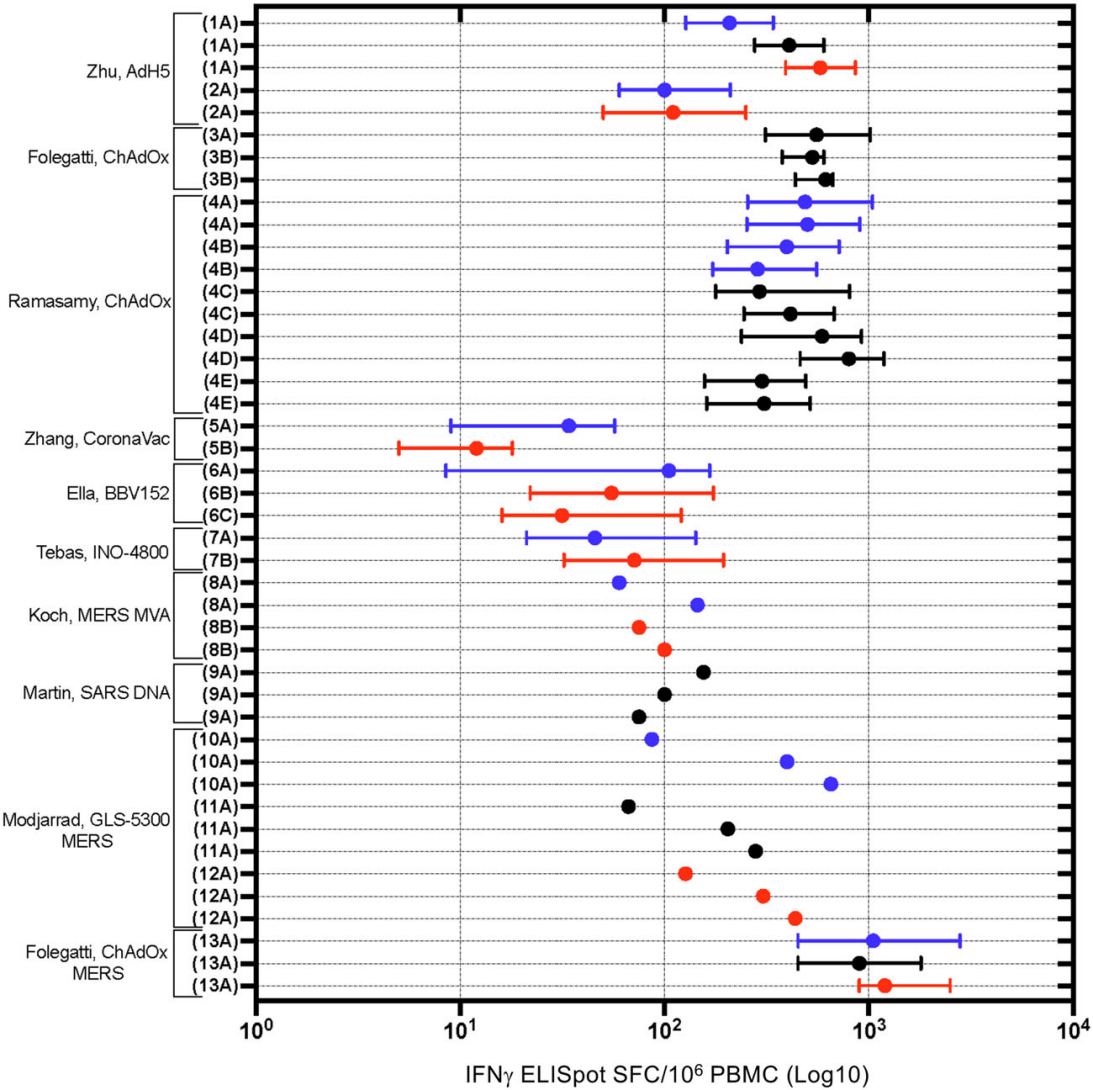
Forest plot of IFNγ ELISpot T cell response following HCoV vaccination in human studies. Blue = low; black = intermediate; red = high dose of vaccine indicated. IFNγ ELISpot data recorded circa 28 days post injection. Log transformed scale used. Vp viral particles, GMT geometric mean, CI confidence interval, IQR interquartile range. (**1A**) Zhu, AdH-5 Phase 1: low dose—5 × 10^10^ vp, intermediate dose—1 × 10^11^ vp, high dose—1.5 × 10^11^ vp; expressed as IFNy expressing cells/10^6^ PBMC (95% CI); (**2A**) Zhu, AdH-5 Phase 2: low dose—1 × 10^11^ vp, high dose—5 × 10^10^ vp; expressed as IFNy expressing cells/10^6^ PBMC (95% CI); (**3A**, **B**) Folegatti, ChAdOx SARS-CoV-2: unspecified dose—5 × 10^10^ vp; (**A**) Prime only, (**B**) Prime-Boost; expressed as SFC/10^6^ PBMC (95% CI); (**4A**–**E**) Ramasamy, ChAdOx: low dose—2.2 × 10^10^ vp, unspecified dose—3.5–6.5 × 10^10^ vp; (**A**) Prime-Boost, 56–69 y/o (low), (**B**) Prime-Boost, ≥70 y/o (low), (**C**) Prime-Boost, 18–55 y/o (unspecified), (**D**) Prime-Boost, 56–69 y/o (unspecified), (**E**) Prime-Boost, ≥70 y/o (unspecified); expressed as SFC/10^6^ PBMC (IQR); (**5A**, **B**) Zhang, CoronaVac, Phase 1: low dose—3 µg, high dose—6 µg; (**A**) post boost (low), (**B**) post boost (high); expressed as SFC/10^6^ PBMC (95% CI); (**6A**–**C**) Ella, BBV152 Phase 1: low dose—3 µg, high dose—6 µg; (**A**) post prime (low + Algel-IMDG), (**B**) post prime (high + Algel-IMDG), (**C**) post prime (high + Algel); expressed as SFC/10^6^ PBMC (IQR); (**7A**, **B**) Tebas, INO-4800: low dose—1.0 mg, high dose—2.0 mg; Boost in a Prime-Boost; (**A**) low dose, (**B**) high dose; expressed as IFNy expressing cells/10^6^ PBMC (median, IQR); (**8A**, **B**) Koch, MVA-MERS-CoV: (**A**) low dos—1 × 10^7^ PFU, (**B**) high dose—1 × 10^8^ PFU; Expressed as SFC/10^6^ PBMC (95% CI); (**9A**) Martin, DNA-SARS-CoV-1: unspecified dose—4 mg; Prime-Boost-Boost; expressed as SFC/10^6^ PBMC; (**10A**) Modjarrad, GLS-500-MERS-CoV: low dose—0.67 mg; Prime-Boost-Boost; expressed as SFC/10^6^ PBMC (95% CI); (**11A**) Modjarrad, GLS-500-MERS-CoV: intermediate dose— 2 mg; Prime-Boost-Boost; expressed as SFC/10^6^ PBMC (95% CI); (**12A**) Modjarrad, GLS-500-MERS-CoV: high dose—6 mg; Prime-Boost-Boost; expressed as SFC/10^6^ PBMC (95% CI); (**13A**) Folegatti, ChAdOx-MERS-CoV: low dose—5 × 10^9^ vp, intermediate dose—2.5 × 10^10^ vp, high dose—5 × 10^10^ vp; expressed as SFC/10^6^ PBMC (95% CI).

T cell responses were evaluated using T cell ELISpot IFNγ responses and/or ICS. Ten human studies evaluated T cell ELISpot IFNγ responses (Fig. 8). The highest IFNγ ELISpot responses were seen in the Folegatti^23^, ChAdOx-MERS-CoV and the Ramasamy^20^, ChAdOx-SARS-CoV-2 vaccine studies. The Zhang^34^, CoronaVac-SARS-CoV-2 vaccine study showed somewhat lower IFNγ SFC. Limited analysis of mean IFNγ ELISpot responses of other trials demonstrated dose-dependent increases in responses in all groups of the Modjarrad, DNA-MERS-CoV^45^ and Koch, MVA-MERS-CoV^42^ vaccines. Overall the log-transformed mean IFNγ ELISpot cell response in the human studies was 2.3 with a range of 2.3. Similar responses were obtained in NHP studies (Supplementary Fig. 3D). As had been seen for the nAb response, the IFNγ ELISpot response to a single 60 µg dose of the Sahin BNT162b1^30^ vaccine was below the lower limit of detection in the majority of vaccinees at d 29.

ICS used to characterize T cell cytokine responses and subsets was described in 14 human studies (Supplementary Table 13). While it is hard to compare effector cell frequencies directly between ELISpot and ICS platforms (since the latter encompass diverse strategies for co-stimulation, peptide panels, gating and cytokine readouts), it can nevertheless be seen that very high responder frequencies are achieved, often around 0.2% of gated CD4^+^ cells being cytokine-positive on stimulation; see for example Jackson mRNA-1273-SARS-CoV-2^24^ intermediate dose, or the Keech NVX-CoV2373^39^ Phase 2 trial data. Cytokine-positive CD4^+^ T cell responses were more prominent than CD8^+^ T cell responses in most studies and IFNγ^+^ expression was the most common T cell effector function recorded in human studies. There was little evidence for Th2 cytokine profiles with Th1 bias most prevalent, although some subjects in Keech, NVX-CoV2373^39^ make a detectable IL-5/IL-13 response.

Five NHP studies evaluated T cell subset responses by ICS. CD4^+^ and CD8^+^ subset responses were stimulated in all studies (Supplementary Table 13). CD8^+^ T cells were the most prominent subset stimulated in NHP trials, in contrast to the findings in human studies. The Yu, DNA-SARS-CoV-2^54^ vaccine elicited minimal CD4/8^+^ IL-4 responses relative to the CD8/4^+^IFNγ responses, indicative of a Th1/Tc1 bias; other NHP studies also recorded Th1/Tc1 responses following vaccination^46,48^. As in the human data, IFNγ^+^ effector function was most prominent in NHP studies (Supplementary Table 13). No significant Th2 responses were recorded following vaccination in any NHP study. The Muthumani, DNA-MERS-CoV^56^ and Corbett, mRNA-1273-SARS-CoV^48^ NHP vaccine groups exhibited dose-dependent increase in T cell cytokine responses.

### Efficacy

Five SARS-CoV-2 human Phase 3 trials reported vaccine efficacy^17,21,22,27,31^ (Supplementary Table 7). Polack, BNT162b2^31^, Baden, mRNA-1273^27^ and Logunov, rAd26r/Ad5^17^ achieved remarkably high efficacy, 94.6%, 94.1% and 91.6%, respectively. Using pooled data from the UK and Brazil Voysey, ChAdOx^21^ reported an efficacy of 70.4%. Follow-up analysis of pooled data from the UK, South Africa and Brazil, Voysey, ChAdOx^22^ reported an efficacy of 66.7% (Supplementary Table 7).

Of 23 NHP studies (Table 1 and Supplementary Tables 4 and 5), 15 evaluate vaccine efficacy. Eleven evaluated viral load by RT-PCR and six measured nAb post-challenge; challenge doses varied greatly. All studies reported a reduction in peak viral load (Log10) compared to control groups. Peak viral load was significantly higher in control groups compared to vaccinated groups post-challenge. Each reported significant protection including, where indicated, from lung pathology/pneumonia, though none attain sterilizing immunity except for the Gao PiCoVacc^50^ inactivated whole virus vaccine given as weekly doses for 3-weeks and in the Guebre-Xabier NVX-CoV2373 vaccine^51^. A caveat of NHP challenge studies is the short time interval between immunization and challenge, 1–6 weeks being most common.

## Discussion

The benefits of comparative evaluation of datasets within the context of a systematic review and meta-analysis are self-evident at a time when enthusiasm for news of vaccine ‘breakthroughs’ can lead to incomplete reports in absence of opportunity for careful, granular, data-led comparison. We considered datasets with respect to binding and neutralizing Ab, prior anti-vector Ab, T cell responses and protection up to March 22, 2021. In doing so, it is a given that the correlates of protection (CoP) for COVID-19—which immune parameter/value gives the clearest actual indicator of protection—are not agreed, though evidence is accruing for nAb titer as a key CoP^46,54,69^.

Safety evaluation has encompassed a range of potential concerns, from initial reactogenicity profile to downstream post-challenge ADE or type 2 lung pathology. The meta-analysis shows a relatively low incidence of local and systemic AE at immunization. It is premature to comment on ADE in SARS-CoV-2 trials and rollout, but thus far it appears not to be an issue. The hypothetical concern originates in macaque studies evaluating either an MVA-vectored vaccine expressing SARS-CoV-1 spike or a SARS-CoV inactivated vaccine, both which resulted in ADE-associated lung immunopathology^70^. An additional mechanism of vaccine-dependent, post-challenge immunopathology is Th2-dependent eosinophilic lung infiltration. Th2 cytokine-dependent lung immunopathology following virus challenge has been noted in vaccines for SARS-CoV-1 and for MERS-CoV in mice, ferrets and NHP^71^. Again, such effects would only become apparent on subsequent exposure to virus, but it is encouraging that T cell responses across all the vaccine platforms compared here show strong skewing to Th1 cytokines, and little Th2 polarization. While anaphylaxis has been a very rare SAE in rollout of the Pfizer-BioNTech BNT162b2 vaccine, this was likely not picked up in the smaller sample size of the Phase 3 trial and with any vaccine allergic individuals excluded^72,73^. European rollout has highlighted rare development of thrombotic thrombocytopenia following ChAdOx1 nCoV-19 and Ad26.COV2.S vaccination^74–76^; this was not a feature identified in Phase III trials.

This review has considered several inactivated virus vaccines, favored for the long track record of the approach (including polio vaccination) and diversity of viral antigens presented, though often considered to elicit less durable immunity. The Lin et al inactivated SAR-CoV1 vaccine elicited high nAb titers with a favourable safety profile, while the similar approach used for inactivation of SARS-CoV-2 also leads to a vaccine which is immunogenic and protective in NHP short-term challenge^44,49^.

Among the many adenoviral-vector based approaches are the ChAdOx, AdH-5 and AdH26/5 vectored vaccines. ChAdOx vectored vaccines have been clinically tested over several years and across diverse infections^77–79^, so that there is a very expansive dataset on the ability to elicit robust Ab and, especially, strong T cell responses, including in the elderly and immunosuppressed. The choice of the chimpanzee viral vector to a considerable degree mitigates the confounder of prior Ab to limit the vaccine. Frequencies of virus-specific T cells induced by the ChAdOx vectors appear in most cases considerably higher than mean T cell responses induced by natural infection, though the extent to which this is a direct CoP remains to be seen. Of others considered here, the MVA-MERS-CoV vector vaccine^42^ produced low levels of nAb compared to other candidates. The mRNA-1273-SARS-CoV-2 vaccine elicited the highest Ab responses of any vaccine after a single dose, with a significant increase upon boosting^24,25^. Th1 cytokine responses were also strong, though requiring a boost. The Folegatti, ChAdOX-SARS-CoV-2 vaccine elicited strong Ab and T cell responses after a single injection, increasing further after boost^19^. At present, there is no data to suggest that Th2 immunopathology will be a significant risk factor for SARS-CoV-2 vaccines. However, ongoing scrutiny will be needed to exclude the possible risk of Th2 immunopathology.

The finding that some vaccines require a second boost for optimal responses while others may not is an important factor for decision-making. The global roll-out of effective vaccines demands unprecedented logistics to achieve coverage for a first round, and all the harder for a second round. At the time of writing, Phase 3 trial data are awaited from the Janssen Ad26.COV2.S Covid-19, which encompasses a 1-dose protocol.

Prior immune memory has been a significant hurdle limiting the immunogenicity of adenoviral and other virus-vectored vaccines^80^. Substantial pre-existing nAb responses were identified to the Zhu, AdH-5-SARS-CoV-2 vaccine^14,15^. Approximately 50% of those vaccinated were positive for pre-existing nAb >1:200, associated with a lower response to vaccination. Pre-immunity has implications for the geographic utility of vaccines, as certain regions have higher rates of specific disease which cause pre-immunity^81^.

Rhesus macaques develop pneumonia and other clinical aspects of SARS-CoV-2 like disease^82,83^ as well as Ab and T cell responses similar to that seen in humans. Each of the tested candidates showed promising efficacy in NHP challenge, with caveats that these studies use greatly differing challenge doses making direct comparison difficult, and also that there tends to be a very short interval between priming and challenge. NHP studies, along with human challenge studies, will continue to play a key role for vital points that are hard to deduce from other approaches: ability of vaccines to achieve sterilizing immunity and delineation of CoP^84^.

It was difficult to attain across the board comparison of T cell immunity due to the different assays reported. Similarly, a lack of standardised antibody binding and neutralizing antibody testing means cross-comparison of binding antibody and nAb titers is limited by inter-assay variability. Efforts by the World Health Organisation to develop an international standard for SARS-CoV-2 neutralization assay calibration may go some way towards resolving this issue^85^. Across vaccine platforms there are trade-offs between Ab titer, T cell frequency, reactogenicity and efficacy, with no single platform uniformly outstanding. RNA-based approaches have proved highly successful, though they can show a disadvantageous AE profile at higher doses and require a boost for maximal efficacy. The adenovirus-based approaches, especially ChAdOx-SARS-CoV-2, are of interest for their ability to induce exceptionally high T cell responses, probably higher than from natural infection, though this may prove less decisive if it transpires that nAb titer is of overriding significance as a CoP. NVX-CoV2373 strategy for spike protein with adjuvant bodes well for a simple approach to attaining exceptionally high, effective high nAb titers and strong protection in NHP challenge studies^39,51^.

There were limitations inherent in this study: while new SARS-CoV-2 vaccine data is being produced rapidly, we have had to impose a finite search window, excluding some candidates that have received attention from press releases and preprints. Gaps in reporting of precise data in some studies prevented the completion of an effective meta-regression analysis of study metrics and determinants of vaccine safety, immunogenicity and efficacy. The lack of control groups in some human trials resulted in a limited meta-analysis of AE.

At a time when we have already witnessed the successful development and roll-out of several COVID-19 vaccines, this review of 23 NHP and 32 human studies offers a template for high-granularity appraisal of the detailed metrics. To fully match the immune studies to fine-tuning of efficacy will require a better understanding of the CoP, particularly, relative importance of nAb and CD4 + responses. However, the front-running vaccines show excellent induction of nAb and T cell responses, and the associated ability to substantially limit severe disease, hospitalisation and death.

Vaccine efficacy against emerging variant strains may, however, be diminished necessitating that vaccines be adapted to act specifically against variant strains and rapidly rolled out. There may also be opportunities to develop heterologous prime boost immunization schedules across vaccine platforms, potentially offering better immune responses and protection. The emergence of variant strains makes rapid mass rollout of high efficacy vaccines essential to reduce any selective advantage. Furthermore, as contingency plans develop, addressing the possible requirement for periodic boosters priming immunity targeted to variant sequences, it will be ever more important to have facilitated protocols that can intersperse use of different platforms, minimizing potential for limitation by anti-vector immunity.

## Methods

### Search strategy & eligibility criteria

This systematic review and meta-analysis were conducted in line with PRISMA guidelines^86^. An electronic search of PubMed and EMBASE databases was carried out using search terms and phrases related to HCoV vaccine candidates (Supplementary Table S1). Inclusion and exclusion criteria were defined to ensure all relevant studies were identified (Supplementary Table S2). Studies or trials evaluating the safety, immunogenicity or efficacy of a coronavirus vaccine candidate in humans or NHP were included. The need to impose a defined search period and also to exclude non-peer-reviewed preprints means that there are a large number of known vaccine candidates in Phase I/II or III trials that have been widely discussed in the press, but are not included in this review. The data included is updated to March 22, 2021 This study is registered with PROSPERO: CRD4202019030.

### Study selection & data extraction

Study selection was performed following pre-determined inclusion and exclusion criteria as described (Fig. 1 and Supplementary Table 2) extracting vaccine characteristics: platform, insert, route of administration, doses and schedule. Characteristics of challenge in NHP studies were also recorded: challenge virus, dose and swab location. For clarity in the Results and Discussion, we discuss specific, named studies by referring to the first author of the study and the shorthand name for the tested vaccine (Table 1 and Supplementary Table 5). Data on local and systemic AE for each of the studies were obtained.

Geometric mean titer (GMT) by enzyme-linked immunosorbent assay (ELISA; IgG, IgG subtypes or IgA) and neutralization assays were extracted. Mean spot-forming cells per million peripheral blood mononuclear cells (SFC/million PBMC) were extracted for T cell ELISpots. If numerical Ab or T cell response datasets were not available, estimates were derived from respective graphs. Intracellular cytokine staining (ICS) data was obtained to analyze T cell responses, where described. Regarding vaccine efficacy analysis from NHP studies, challenge dose, viral strain, peak viral load, reverse transcription polymerase chain reaction (RT-PCR) swab location, RT-PCR positivity data and neutralizing Ab titers post-challenge were collated. To analyze human vaccine efficacy, the efficacy endpoints and overall vaccine efficacy (%) were extracted from relevant studies.

### Data analysis

RevMan 5.2 and GraphPad Prism were used for statistical analysis. With respect to AE analysis, Forest plot analysis was used to look at interstudy heterogeneity for AEs. Results were reported as risk ratios (RR) comparing the incidence of an AEs in experimental groups to control groups. A random effects, Mantel–Hanzeal model (95% CI) was used to determine effect sizes between studies. Statistical heterogeneity was assessed using *I*^2^ statistics: an *I*^2^ value of >50%; *P* < 0.05 was considered to represent severe heterogeneity, *I*^2^ value of 30–50%; *P* < 0.05 was considered to represent moderate heterogeneity and <30%; *P* < 0.05 was deemed to constitute insignificant heterogeneity. Since many first-in-human studies lacked control groups, descriptive analysis of AE was conducted. A grade three AE is here considered a severe AE. Serious AE (Grade 4) recorded in human studies were presented in tabular form.

Due to the existence of multiple vaccine groups in each study, all vaccine groups were considered a statistical unit^87^. Vaccine groups are considered to be experimental and control groups in the human and NHP trials. If a single dose of a vaccine candidate was used, it is defined as an unspecified dose.

To evaluate interstudy Ab responses, we analyzed the GMT and nAb titer from ELISA and neutralizing assays respectively. There was a lack of comprehensive numerical datasets of Ab titers across human studies; in some cases Ab titers were estimated from graphical representations. Ab titers analyzed were collected circa 28 d after a given injection, allowing for inclusion of Ab titer data from vaccine regimens involving prime/boosts. Transformed mean log Ab titers (Log10) were estimated from human and NHP studies to determine statistical difference between groups using an unpaired *t* test (95% CI).

T cell responses were evaluated by extracting IFNγ ELISpot data. In some cases, estimated frequencies were extracted from graphs. T cell responses were extracted circa 28 d after individual immunizations and represented by Forest plot to show interstudy differences in ELISpot SFC/million PBMC. Transformed log mean T cell responses were obtained from human and NHP studies to test statistical differences between NHP and human groups.

For NHP efficacy analysis, we analyzed differences between control and experimental peak viral loads and nAb responses (Log10) post-challenge to examine vaccine-induced protection.

### Reporting summary

Further information on research design is available in the Nature Research Reporting Summary linked to this article.

## Supplementary information

Supplementary Information File

Reporting Summary

## Data Availability

All data generated or analyzed during this study are included in the published article (and Supplementary Information files).

## Code availability

This study did not use custom code.
