# Supplementary material for: Comparative systematic review and meta-analysis of reactogenicity, immunogenicity and efficacy of vaccines against SARS-CoV-2
Source: NPJ Vaccines. 2021 May 13;6:74. doi: 10.1038/s41541-021-00336-1 (PMC8116645; doi:10.1038/s41541-021-00336-1)
Supplement: Supplementary file 1 — Supplementary Information File [file 41541_2021_336_MOESM1_ESM.pdf]

Supplementary Information File

Comparative systematic review and meta-analysis of reactogenicity, immunogenicity and efficacy of vaccines against SARS-CoV-2

Ian McDonald[1], Sam M Murray[1], Catherine J Reynolds[1], Daniel M Altmann[2]* and Rosemary J Boyton[1,3]*

[1]Department of Infectious Disease, Faculty of Medicine, Imperial College London, UK

[2]Department of Immunology and Inflammation, Faculty of Medicine, Imperial College London, UK

[3]Lung Division, Royal Brompton and Harefield Hospitals, London, UK

*Address for correspondence:

Prof. Rosemary Boyton, PhD, FRCP, FHEA

Lung Immunology Group, Adult Infectious Disease, Department of Infectious Disease, Faculty of Medicine, Room 8N22 Commonwealth Building, Hammersmith Hospital Campus,

Imperial College London, Du Cane Road, London W12 0NN, UK

Email: r.boyton@imperial.ac.uk

&

Prof. Daniel Altmann, PhD

Department of Immunology and Inflammation, Faculty of Medicine,

Imperial College London, Du Cane Road, London W12 0NN, UK

Email: d.altmann@imperial.ac.uk

Supplementary Figure 1: Heat map and table showing countries where HCoV pre-clinical and clinical studies were conducted

Supplementary Figure 2: Percent of Grade 3 adverse events (AE) recorded in human trials

Supplementary Figure 3: Human (n=25) and NHP (n=23) studies; comparative analysis of immunological readouts

Supplementary Table 1: Database Search Strategy: Search terms, phrases and limits related to HCoV vaccine research used in accordance with PRISMA guidelines

Supplementary Table 2: Inclusion and exclusion criteria

Supplementary Table 3: Characteristics of the human studies

Supplementary Table 4: Characteristics of the NHP studies

Supplementary Table 5. Summary of vaccine studies in NHP

Supplementary Table 6: Demographic characteristics of the human studies

Supplementary Table 7: Vaccine efficacy against COVID-19

Supplementary Table 8: NHP safety analysis

Supplementary Table 9. Interstudy analysis of individual local adverse events reported in vaccine group (excluding control group) of coronavirus vaccine studies.

Supplementary Table 10: Interstudy analysis of individual systematic adverse events reported in vaccine group (excluding control group) of coronavirus vaccine studies.

Supplementary Table 11: Serious adverse events

Supplementary Table 12: Pre-existing anti-vector neutralising antibody titers recorded before vaccination and their correlation with anti-RBD seroconversion or SARS-CoV-2 neutralization in adenoviral vectored SARS-CoV-2 vaccines

Supplementary Table 13. Human and NHP T cell analysis

Supplementary Figure 1:Heat map and table showing countries where HCoV pre-clinical and clinical studies were conducted

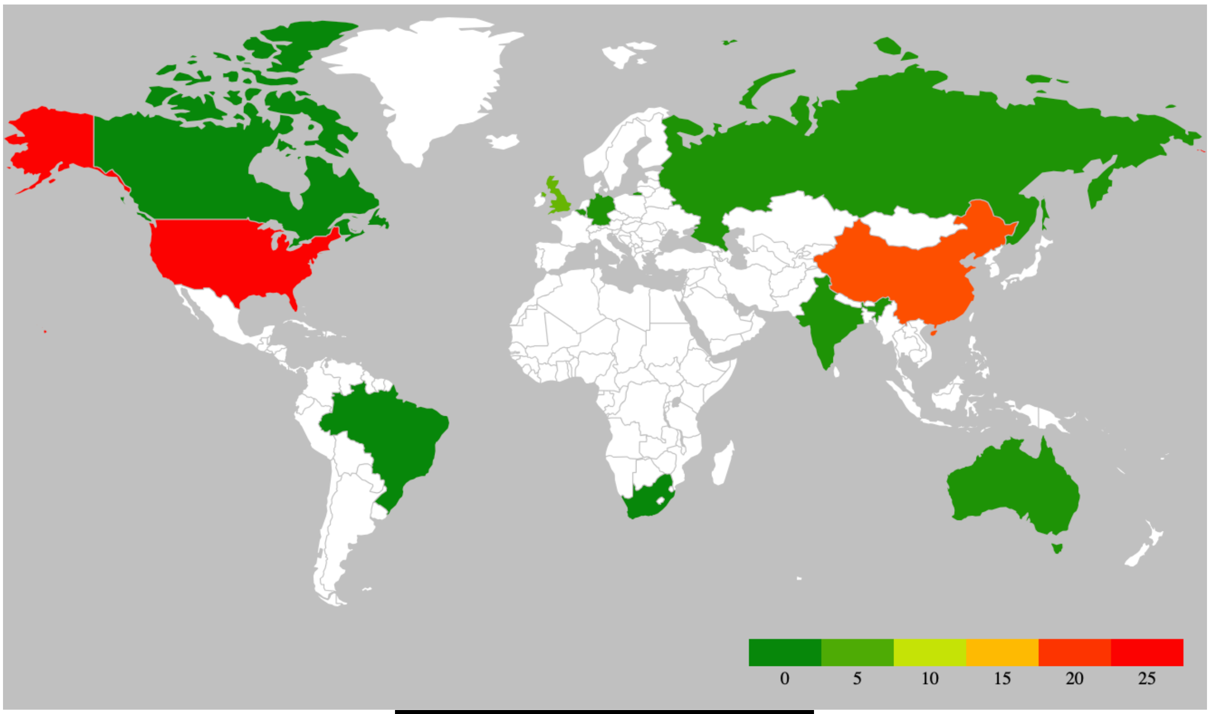

Country	Number
United States	21
China	18
United Kingdom	5
Belgium	2
Germany	2
India	2
Australia	2
Russia	2
Canada	1
Brazil	1
South Africa	1

Supplementary Figure 2. Percent of Grade 3 adverse events (AE) recorded in human trials. Grade 3 AE in both experimental and placebo groups are included in this analysis. First author, vaccine platform and number of participants in each study shown on the x-axis. Zhu, AdH5 (1): AdH5-SARS-CoV-2 phase 1 trial, Zhu, AdH5 (2): AdH5-SARS-CoV-2 phase 2 trial, Folegatti, ChAdOx1 (1): ChAdOx-MERS.

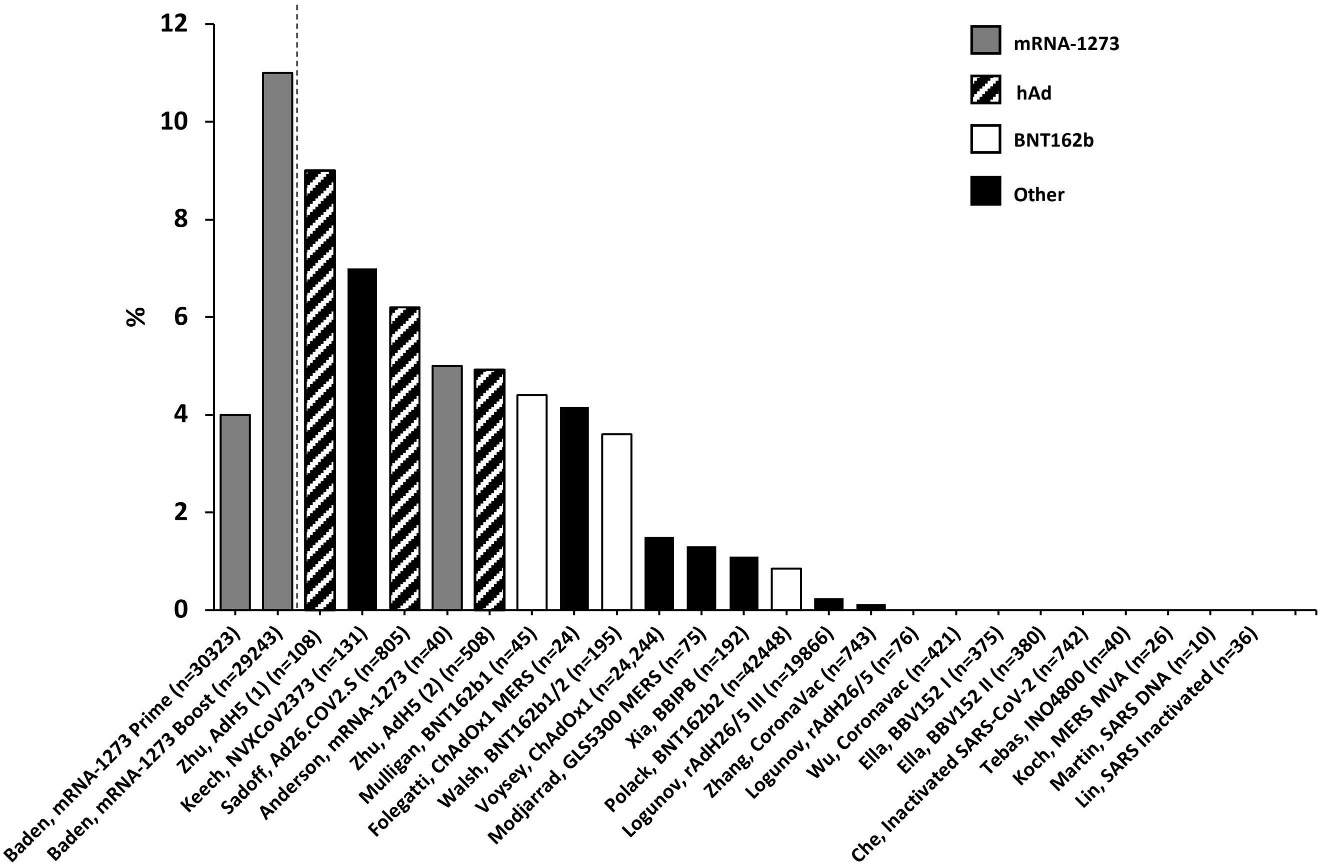

Supplementary Figure 3. Human (n=25) and NHP (n=23) studies; comparative analysis of immunological readouts: (A) ELISA-IgG, (B) Live neutralization antibody assay, (C) Pseudovirus neutralization antibody assay, (D) ELISpot T cell response. Data are log transformed. Unpaired t-test of independence used to evaluate the statistical difference between human and NHP vaccine group responses (p<0.05 was taken to indicate a significant difference)

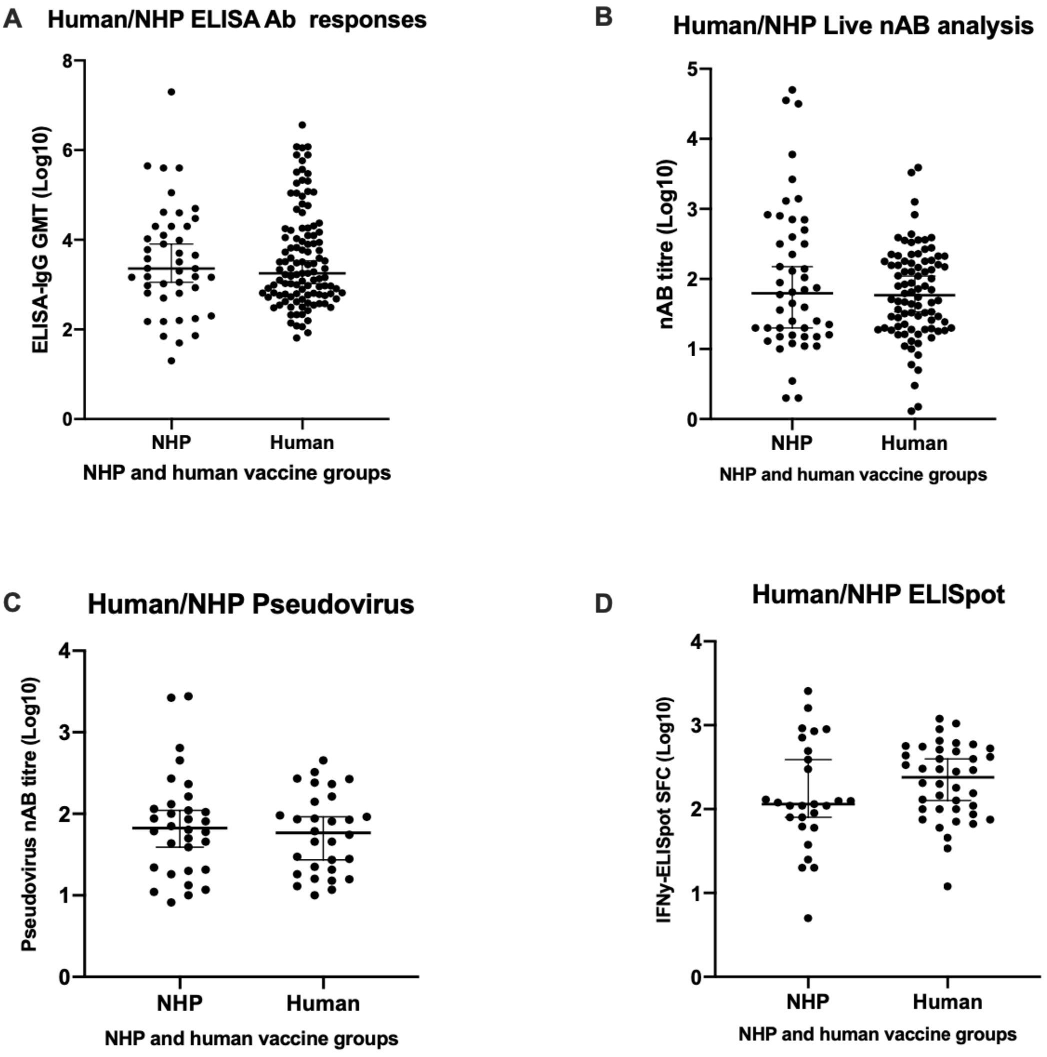

Supplementary Table 1:Database Search Strategy: Search terms, phrases and limits related to HCoV vaccine research used in accordance with PRISMA guidelines.

Data base	Search strategy
PUBMED	(Coronavirus) OR ("sars"[All Fields])) OR ("middle east respiratory syndrome"[All Fields])) OR ("sars"[Title/Abstract]) OR ("mers"[Title/Abstract])) AND (Vaccin*)) NOT (Review[Publication Type]) [Filter – Year of Publication – 2003-2021]
EMBASE	(coronaviridae OR 'severe acute respiratory syndrome' OR 'middle east respiratory syndrome coronavirus' OR 'sars' OR 'mers') AND (vaccin*) [Filter – Year of Publication – 2003-2021]

Supplementary Table 2: Inclusion and exclusion criteria

Inclusion Criteria

- Any study evaluating the safety, immunogenicity or efficacy of a coronavirus vaccine candidate in NHP or humans

Exclusion Criteria

- *In vitro* studies
- Other animal models
- Reviews, editorials, conference proceedings
- Unrelated to coronavirus vaccine development
- Duplicates

Note on exclusion of Pre-Prints from this analysis

- We have only included peer reviewed data in this analysis

Supplementary Table 3: Characteristics of the human studies

Study ID<br>Location<br>Ref No	Study Design,<br>Length,<br>Population (N),<br>Age	Pathogen	Vaccine Platform	Antigen Insert	Group + Doses,<br>Boosting,<br>Route of Administration,	Measure of Ab Response	Measure of Cellular Response + Days
Zhu et al,<br>(14)<br>2020<br>China	Safety, Tolerability and Immunogenicity.<br>Open-Label, Dose-Escalation, Non-Randomized Phase 1 trial<br>4 Weeks<br>N=108<br>18-60 y/o	SARS-CoV-2	AdH5	Full-Length<br>SARS-CoV-2<br>Spike GP	Low Dose = 5x10^{10}vp (N=36)<br>Intermediate Dose = 1x10^{11}vp (N=36)<br>High Dose = 1.5x10^{11}vp (N=36)<br>Not Boosted<br>IM	**ELISA-IgG**<br>D14-D28<br>**Live nAb Assay**<br>D14-D28	**IFNγ Linked ELISpot**<br>D0-D28<br>**Flow cytometry/ICS**<br>D0-D28
Zhu et al,<br>(15)<br>2020<br>China	Safety and Immunogenicity<br>A randomized, double-blind, placebo-controlled trial<br>Phase 2<br>4 Weeks<br>N=508	SARS-CoV-2	AdH5	Full-Length<br>SARS-CoV-2<br>Spike GP	Low dose = 1x10^{11} vp (N=253)<br>High Dose = 5x10^{10}vp (N=129)<br>Placebo = Vaccine excipients (N=126)<br>Not boosted<br>IM	**ELISA-IgG**<br>D0-D28<br>**Live nAb assay**<br>D0-D28<br>**VSV-pseudovirus nAb assay**<br>D0-D28	**IFNγ -ELISpot**<br>D0-D28
Logunov et al<br>(16)<br>2020<br>Russia	Safety and Immunogenicity<br>Open label non-randomized phase 1/2 trial<br>7 weeks<br>N=76<br>18-60y/o	SARS-CoV-2	rAd26 and<br>rAd5<br>Gam-COVID<br>'Sputnik'	Full-Length<br>SARS-CoV-2<br>Spike GP	**Gam-COVID-Vac**<br>Group 1= Ad26 (N=9)<br>Group 2= Ad5: (N=9)<br>Group 3= Ad26+Ad5: (N=20)<br>**Gam-COVID-Lyo**<br>Group 4= Ad26: (N=9)<br>Group 5= Ad5: (N=9)<br>Group 6= Ad26+Ad5: (N=20)<br>Dosage = 1x10^{11}<br>Group 3 and 6 boosted D21<br>IM	**ELISA-IgG**<br>D0-D14-D21-D28-D42<br>**MNA_{50}**<br>D0-D14-D21-D28-D42	**IFNγLinked ELISpot**<br>D0-D14-D28<br>**Flow cytometry/ICS**<br>D0-D14-D28
Logunov et al,<br>(17)<br>2021<br>Russia	Safety, efficacy<br>Phase 3, randomized, double-blind, placebo-controlled, multi-center<br>N= 21,977<br>>18y/o	SARS-CoV-2	rAd26 and<br>rAd5<br>Gam-COVID<br>'Sputnik'	Full-Length<br>SARS-CoV-2<br>Spike GP	Vaccine N = 14964<br>Placebo N =4902<br>All boosted D21<br>IM	**ELISA-IgG N=456**<br>D42<br>**$TCID_{50}$ N=100**<br>D42	**IFNγ ELISA n=58**<br>D1, D28
Sadoff et al,<br>(18)<br>2021<br>Belgium/USA	Safety, immunogenicity<br>Phase 1/2a, randomized, double-blind multi-center, placebo-controlled<br>Ongoing<br>N=805<br>>18y/o	SARS-CoV-2	rAd26.COV2.S	Full-length<br>stabilized<br>SARS-CoV-2<br>spike GP	Low dose = 5x10^{10} VP (N=323)<br>High dose = 1x10^{11} VP (N=318)<br>Placebo=N=16<br>Boosted D57<br>IM	**ELISA-IgG**<br>D15, 29,57,71<br>**MNA_{50}**<br>D15,29,57,71	**ICS**<br>D1-15
Folegatti et al,<br>(19)<br>2020<br>UK	Safety, Immunogenicity and efficacy<br>Single blinded, multi-center, randomized, Phase1/2 Trial<br>8 Weeks<br>N=1077<br>18=55y/o	SARS-CoV-2	ChAdOx,<br>MenACWY<br>(Control)	Full-Length<br>SARS-CoV-2<br>Spike GP	Single dosage: 5x10^{10}vp<br>Group 1: Single injection (N=44) + control (N=44)<br>Group 2: Single Injection (N=489) + control (N=489)<br>Group 3: Two injections (N=10)<br>Group 4: Single injection (N=489) + control (N=489)<br>Boosted D28<br>IM	**ELISA-IgG**<br>D0-D56<br>**Microneutralization assay ($MNA_{50/80/90}$)**<br>D0-D42<br>**Lentivirus Pseudovirus nAb assay (IC_{50})**<br>D0-D42<br>**$PRNT_{50}$**<br>D0-D28<br>**Marburg nAB assay (IC_{100})**<br>D0-D56<br>**Multiplex Immunoassay**<br>D0-D42	**IFNγ Linked ELISpot**<br>D0-D28

Ramasamy et al<br>(20)<br>2020<br>UK	Safety and Immunogenicity<br>Single blind, randomized Phase 2/3 trial<br>Ongoing<br>N=560<br>≥18y/o	SARS-CoV-2	ChAdOx,<br>MenACWY<br>(Control)	Full-Length<br>SARS-CoV-2<br>Spike GP	Prime, low dose = 2.2x10^{10}vp<br>(N=100)<br>Prime, standard dose = 3.5-6.5x10^{10}vp<br>(N=100)<br>Prime-Boost, low dose = 2.2x10^{10}vp<br>(N=195)<br>Prime-Boost, standard dose = 3.5-6.5x10^{10}vp<br>(N=157)	**ELISA-IgG**<br>D0-D28-D42-D56<br>**SEAP Neutralization assay**<br>D0-D28-D42-D56<br>**Microneutralization assay (MNA_{80})**<br>D0-D28-D42-D56	**IFNγ Linked ELISpot**<br>D0-D14-D28-D42
Voysey et al<br>(21)<br>2020<br>UK, Brazil,<br>South Africa	Safety and Efficacy<br>Blinded, randomized controlled trial<br>Ongoing<br>N=23,848 (11,636 included in analysis)<br>≥18y/o	SARS-CoV-2	ChAdOx,<br>MenACWY<br>(Control)	Full-Length<br>SARS-CoV-2<br>Spike GP	Experimental groups= 2.2 to 6.5x10^{10}<br>(N=4440)<br>Control groups= MenACWY<br>(N=4455)<br>Boost up to D84<br>IM	NA	NA
Voysey et al,<br>(22)<br>2021<br>UK/South Africa/Brazil	Immunogenicity, efficacy<br>Pooled analysis of Phase 1/2 & 2/3(UK), Phase 3 (Brazil), Phase 2/3 SA<br>N= 24,422<br>18-55, ≥18, 18-65y/o	SARS-CoV-2	ChAdOx,<br>MenACWY<br>(Control)	Full-Length<br>SARS-CoV-2<br>Spike GP	Phase 1/2 UK<br>Vaccine N=544<br>Placebo N=533<br>Phase 2/3 UK<br>Vaccine N=5593<br>Placebo N=5211<br>Phase 3 Brazil<br>Vaccine N=5207<br>Placebo N=5209<br>Phase 2/3 SA<br>Vaccine N=1064<br>Placebo N=1061<br>Boost intervals:<br><6 weeks N=7746<br>6-8 weeks N=2121<br>9-11 weeks N=1864<br>≥12 weeks N=2649<br>IM	**ELISA-IgG**<br>D0, D56, D84, D112, D140, D182 (N=44), D28 after 2nd dose (N=3337)<br>**Pseudotype nAb assay (IC_{50})**<br>D28 after 2nd dose.<br>(N=893)	NA
Folegatti et al,<br>(23)<br>2020<br>UK	Safety and Immunogenicity,<br>Open-Label, Dose-Escalation, Non-Randomized, Uncontrolled Phase 1 trial<br>52 Weeks<br>N=24<br>18-55 y/o	MERS-CoV	ChAdOx1	Full-Length<br>MERS-CoV<br>Spike GP	Low Dose = 5x10^{9}vp (N=6)<br>Intermediate Dose = 2.5x10^{10}vp (N=9)<br>High Dose = 5x10^{10}vp (N=9)<br>Not Boosted<br>IM	**ELISA-IgG**<br>D0-D364<br>**Live nAb Assay**<br>D0-D28<br>**HuH-7 Pseudotyped nAb Assay**<br>D0-D28	**IFNγ Linked ELISpot**<br>D0-D364
Jackson et al,<br>(24)<br>2020<br>USA	Safety and Immunogenicity,<br>Open-Label, Non-Randomized,<br>Phase 1 trial<br>8 Weeks<br>N=45<br>18-55 y/o	SARS-CoV-2	RNA<br>mRNA-1273<br>(LNP)	SARS-CoV-2<br>Spike S2-P	Low Dose = 25ug (N=15)<br>Intermediate = 100ug (N=15)<br>High = 250ug (N=15)<br>Boosted D29<br>IM	**ELISA-IgG**<br>D1-D57<br>**$PRNT_{80}$**<br>D1-D43<br>**Lentivirus Pseudovirus nAb assay (IC_{50})**<br>D1-D57	**Flow cytometry/ICS**<br>D1-D29
Anderson et al<br>(25)<br>2020<br>USA	Safety and Immunogenicity<br>Open-label, dose-escalation, phase 1 trial<br>Ongoing<br>N=40<br>>56y/o	SARS-CoV-2	mRNA-1273<br>(LNP)	SARS-CoV-2<br>Spike S2-P	**56-70y/o**<br>Group 1 = 25ug (N=10)<br>Group 2 = 100ug (N=10)<br>**≥71y/o**<br>Group 3 = 25ug (N=10)<br>Group 4 = 100ug (N=10)<br>All Boosted D29<br>IM	**ELISA-IgG**<br>D1-D15-D29-D36-D43-D57<br>**Lentivirus Pseudovirus nAb assay**<br>D1-D15-D29-D36-D43-D57<br>**HTNA**<br>D1-D29-D43<br>**FRNT**<br>D1-D29-D43<br>**PRNT**<br>D1-D29-D43	**Flow Cytometry/ICS**<br>D1-D29-D43
Chu et al,<br>(26)<br>2021<br>USA	Safety, immunogenicity<br>Phase 2, randomized, observer-blind, placebo-controlled, multi-center<br>N=600<br>18-55y/o (N=300), ≥55y/o (N=300)	SARS-CoV-2	mRNA-1273<br>(LNP)	SARS-CoV-2<br>Spike S2-P	50ug N=200<br>100ug N=200<br>Placebo N=200<br>All boosted D29<br>IM	**ELISA-IgG**<br>D0,29,43,57<br>**Live virus microneutralization assay (MN_{50})**<br>D0,29,43,57	NA

Baden et al<br>(27)<br>2020<br>USA	Safety and Efficacy<br>Phase 3 randomized, observer-blinded, placebo-controlled trial<br>Ongoing<br>N=30,420	SARS-CoV-2	mRNA-1273<br>(LNP)	SARS-CoV-2<br>Spike S2-P	Experimental = 100ug (N=15,181)<br>All Boosted (D28)<br>Placebo = Saline (N=15,170)	NA	NA
Walsh et al<br>(28)<br>2020<br>USA	Safety and Immunogenicity<br>Single-blinded, dose escalation, placebo-controlled phase 1 trial<br>Ongoing<br>N=195<br>18-55y/o, 65-85y/o	SARS-CoV-2	LNP mRNA<br>BNT162b1<br>and<br>BNT162b2	BNT162b1:<br>Trimerized<br>RBD<br>BNT162b2:<br>Membrane-anchored, full length, perfusion stabilized spike protein	**BNT162b1 (18-55y/o)**<br>Group 1 =10ug (N=12)<br>Group 2 =20ug (N=12)<br>Group 3 =30ug (N=12)<br>Group 4 =100ug (N=12)<br>Group 5 = Placebo (N=12)<br>**BNT162b1 (65-85y/o)**<br>Group 1 =10ug (N=12)<br>Group 2 =20ug (N=12)<br>Group 3 =30ug (N=12)<br>Group 4 = Placebo (N=9)<br>**BNT162b2 (18-55y/o)**<br>Group 1 =10ug (N=12)<br>Group 2 =20ug (N=12)<br>Group 3 =30ug (N=12)<br>Group 4 = Placebo (N=9)<br>**BNT162b2 (65-85y/o)**<br>Group 1 =10ug (N=12)<br>Group 2 =20ug (N=12)<br>Group 3 =30ug (N=12)<br>Group 4 = Placebo (N=9)<br>All boosted D21<br>IM	**WT serum neutralization assay**<br>D1-D21-D28-D35<br>**S1 IgG direct Luminex immunoassay**<br>D1-D21-D28-D35<br>**RBD IgG direct Luminex immunoassay**<br>D1-D21-D28-D35	**NA**
Mulligan et al<br>(29)<br>2020<br>USA	Safety, Tolerability and Immunogenicity<br>Randomized, single-blind, dose escalation, placebo-controlled phase ½ trial<br>Ongoing<br>N=45<br>18-55y/o	SARS-CoV-2	LNP mRNA<br>BNT162b1	BNT162b1:<br>Trimerized<br>RBD	Group 1= 10ug (N=12)<br>Group 2= 30ug (N=12)<br>Group 3= 100ug (N=12)<br>Group 4 = Placebo (N=9)<br>Group 1/2 boosted D21<br>IM	**RBD IgG direct Luminex immunoassay**<br>D7-D21-D28-D35<br>**VNT_{50}**<br>D7-D21-D28-D35	**NA**
Sahin et al<br>(30)<br>2020<br>Germany	Safety, tolerability and Immunogenicity<br>Open label, dose escalation phase 1 trial<br>7 weeks<br>N=60<br>18-85y/o	SARS-CoV-2	LNP mRNA<br>BNT162b1	BNT162b1:<br>Trimerized<br>RBD	Group 1 =1ug (N=12)<br>Group 2 =10ug (N=12)<br>Group 3 =30ug (N=12)<br>Group 4 =50ug (N=12)<br>Group 5 =60ug (N=12)<br>All Boosted D22<br>IM	**RBD IgG direct Luminex immunoassay**<br>D1-D8-D22-D29-D43<br>**VNT_{50}**<br>D1-D8-D22-D29-D43<br>**VSV-pseudovirus nAb assay**<br>D1-D8-D22-D29-D43	**IFNγ Linked ELISpot**<br>D1-D29<br>**Flow cytometry/ICS**<br>D1-D29
Polack et al<br>(31)<br>2020<br>USA	Safety and Efficacy<br>Multinational, observer blinded, placebo-controlled Phase 3 trial<br>Ongoing<br>N=43,448<br>>16 y/o	SARS-CoV-2	LNP mRNA<br>BNT162b2	Prefusion stabilised full-Length SARS-CoV-2 Spike GP	Experimental = 30ug (N=21720)<br>Placebo = Saline (N=21728)<br>All Boosted (D21)<br>IM	NA	NA
Che et al<br>(32)<br>2020<br>China	Safety and Immunogenicity<br>Double-blind, randomized phase 2 trial<br>7 weeks<br>N=742<br>18-59y/o	SARS-CoV-2	Virus Inactivated by Formaldehyde and ß-Propiolactone	Inactivated SARS-CoV-2 Virus (KMS-1 Strain)	Medium dose = 100 EU viral antigen + Alum= (N=300)<br>High dose = 150 EU viral antigen + Alum (N=300)<br>Placebo = Alum (N=150)<br>All Boosted D14/ 28	**ELISA-IgG**<br>D0-D14-D28<br>**Live nAb Assay**<br>D0-D14-D28	**NA**
Xia et al<br>(33)<br>2020	Safety and Immunogenicity<br>Double-blind, randomized, placebo-controlled phase 1/2 trial	SARS-CoV-2	Virus<br>Inactivated<br>by	Inactivated<br>SARS-CoV-2	**Phase 1**<br>Low dose = 2ug + Alum (N=48)<br>Medium dose = 4ug + Alum (N=48)	**Live nAb Assay**<br>D7-D14-D28-D32-42	**NA**

China	Ongoing<br>Phase 1 - N=192<br>Phase 2 – N=448		ß-<br>Propiolactone<br>(BBIBP-CorV)	Virus (HB02<br>Strain)	High Dose = 8ug + Alum (N=48)<br>Placebo = Saline + Alum (N=47)<br>All Boosted D28<br>**Phase 2**<br>Group 1 = 8ug + Alum (N=112)<br>Group 2 = 4ug + Alum and placebo<br>(N=112), Boosted D14<br>Group 3 = 4ug + Alum and placebo<br>(N=112), Boosted D21<br>Group 4 = 4ug + Alum and placebo<br>(N=112), Boosted D14-D28<br>All used IM		
Zhang et al<br>2020<br>(34)<br>China	Safety, Tolerability and<br>Immunogenicity<br>Double-blind, placebo-controlled<br>Phase 1 and 2 trial<br>Ongoing<br>Phase 1 - N=144<br>Phase 2 - N=600<br>18-59y/o	SARS-CoV-2	Virus<br>Inactivated<br>by<br>ß-<br>Propiolactone<br>(CoronaVac)	Inactivated<br>SARS-CoV-2<br>Virus (CN02<br>Strain)	**Phase 1**<br>Low Dose = 3ug + Alum (N=48)<br>High Dose = 6ug + Alum (N=48)<br>Placebo = Alum (N=48)<br>All Boosted D14<br>IM<br>**Phase 2**<br>Low Dose = 3ug + Alum (N=237)<br>High Dose = 6ug + Alum (N=237)<br>Placebo = Alum (N=120)<br>All Boosted D28<br>IM	**ELISA-IgG/IgM**<br>D0-D28-D42-D56<br>**Microcytopathogenic**<br>**Live nAb Assay**<br>D0-D14-D28<br>**Pseudovirus nAb assay**<br>D0-D14-D28	**IFNγ Linked ELISpot**<br>D0-D14-D28<br>**Flow cytometry/ICS**<br>D0-D14-D28
Wu et al,<br>(35)<br>2021<br>China	Safety, tolerability, immunogenicity<br>Phase 1/2 randomized, double-<br>blind, placebo-controlled<br>Phase 1 N=72 18-59y/o<br>Phase 2 N=350 ≥60y/o	SARS-CoV-2	Virus<br>Inactivated<br>by<br>ß-<br>Propiolactone<br>(CoronaVac)	Inactivated<br>SARS-CoV-2<br>Virus (CN02<br>Strain)	1.5ug Alum N=100<br>3ug Alum N=124<br>6ug Alum N= 124<br>Placebo N= 74<br>All boosted D28<br>IM	**Microcytopathogenic**<br>**Live nAb assay**<br>D0, D56	NA
Ella et al,<br>(36)<br>2021<br>India	Safety, immunogenicity<br>Phase 1 double-blind, multi-center,<br>randomized, placebo-controlled<br>N=375<br>18-55y/o	SARS-CoV-2	BBV152	Inactivated<br>whole<br>virion	3ug with Algel-IMDG (N=100)<br>6ug with Algel-IMDG (N=100)<br>6ug with Algel (N=100)<br>Placebo= Algel only (N=75)<br>Boosted D14<br>IM	**ELISA-IgG**<br>D0, 28<br>**Microneutralization**<br>**assay (MNT_{50})**<br>D0, D28<br>**Plaque-reduction**<br>**neutralization test**<br>**(PRNT50)**	**ICS**<br>D0, 28<br>**IFNγ ELISpot**<br>D0, 28
Ella et al,<br>(37)<br>2021<br>India	Safety, immunogenicity<br>Phase 2, double-blind, randomized,<br>multi-center<br>N= 380<br>12-65y/o	SARS-CoV-2	BBV152	Inactivated<br>whole<br>virion	3ug Algel-IMDG N=190<br>6ug Algel-IMDG N=190<br>Boosted D28<br>IM	**ELISA-IgG**<br>D0, 28,42,56<br>**Microneutralization**<br>**assay (MNT_{50})**<br>D0, 56<br>**Plaque-reduction**<br>**neutralization test**<br>**(PRNT50)**<br>D0, 56	**Luminex ELISA for Th1/2**<br>**cytokines**<br>D0,42<br>**FACS**<br>D0, 104
Xia et al<br>(38)<br>2020<br>China	Safety and Immunogenicity<br>Randomized, double-blind, placebo-<br>controlled phase 1/2 trial<br>Ongoing<br>Phase 1: N=96<br>Phase 2: N=224<br>18-59y/o	SARS-CoV-2	Virus<br>Inactivated<br>by<br>ß-<br>Propiolactone	Inactivated<br>SARS-CoV-2<br>Virus<br>(WIV04<br>Strain)	**Phase 1 (D28,58 boost)**<br>Low dose= 2.5ug (N=24)<br>Medium dose= 5ug (N=24)<br>High dose= 10ug (N=24)<br>Alum only= (N=24)<br>**Phase 2 (D14 boost)**<br>Medium dose= 5ug (N=84)<br>Alum Only= (N=28)<br>**Phase 2 (D21 boost)**<br>Medium dose= 5ug (N=84)<br>Alum Only= (N=28)	**ELISA-IgG**<br>D4-D14-D21-D35-D56-<br>D70<br>**$PRNT_{50}$**<br>D4-D14-D21	
Keech et al<br>(39)<br>2020<br>USA, Australia	Safety and Immunogenicity<br>Randomized and placebo-<br>controlled phase 1/2 trial<br>Ongoing<br>N=131<br>18-59y/o	SARS-CoV-2	Recombinant<br>Nanoparticle<br>NVX-CoV2373	Full-Length<br>SARS-CoV-2<br>Spike GP	Group 1= Placebo (N=23)<br>Group 2= 25ug (N=25)<br>Group 3= 5ug + 50ug Alum (N=26)<br>Group 4= 25ug + 50ug Alum (N=25)<br>Group 5= 25ug + 50ug Alum (N=26)<br>Group 1/2/3/4 boosted D21	**ELISA-IgG**<br>D0-D7-D21-D28-D35<br>**MNA_{99}**<br>D0- D21-D35	**Flow cytometry/ICS**<br>D0-D28

					IM		
Tebas et al<br>(40)<br>2020<br>USA	Safety, tolerability,<br>immunogenicity,<br>Phase 1, open-label, multi-center,<br>Ongoing<br>N=40<br>18-50y/o	SARS-CoV-2	DNA<br>(INO-4800)	SARS-CoV-2<br>spike GP	Low dose = 1mg<br>High dose=2mg<br>Boosted D28<br>ID with electroporation	**ELISA IgG**<br>D7, 42<br>**Authentic virus nAb assay** D7, 42	**IFNγ ELISpot**<br>D7, 42<br>**ICS**<br>D7, 42
Richmond et al,<br>(41)<br>2021<br>Australia	Safety, immunogenicity<br>Phase 1, randomized, double-blind placebo-controlled<br>N=151<br>18-54, 55-75y/o	SARS-CoV-2	S-Trimer<br>(SCB-2019)	Trimer-Tag fused trimerized S protein	3ug w/o Adjuvant n=8<br>3ug AS03 N=16<br>3ug CpG/Alum N=16<br>9ug w/o Adjuvant N=8<br>9ug AS03 N=16<br>9ug CpG/Alum N=16<br>30ug w/o Adjuvant N=9<br>30ug AS03 N=16<br>30ug CpG/Alum N=16<br>Placebo N=30<br>All boosted D21<br>IM	**ELISA-IgG**<br>D1,D22,D36,D50<br>**ACE2-Competitive ELISA**<br>D1,D22,D36,D50<br>**Authentic WT Neutralization**<br>D1,D22,D36,D50	**ICS**<br>D1,D22,D36,D50
Koch et al,<br>(42)<br>2020<br>Germany	Safety and Immunogenicity,<br>Open-Label, Non-Randomized,<br>Phase 1 trial<br>24 Weeks<br>N=26<br>18-55 y/o	MERS-CoV	MVA	Full-Length MERS-CoV Spike GP	Low Dose = $1x10^7$ PFU (N=14)<br>High Dose = $1x10^8$ PFU (N=12)<br>Control = Blood samples from healthy volunteers (N=6)<br>Boost: D28<br>IM	**ELISA-IgG**<br>D0-D180<br>**Live nAb Assay**<br>D0-D180<br>**$PRNT_{80}$**<br>D0-D180	**IFNγ Linked ELISpot**<br>D0-D180<br>**Flow cytometry/ICS**<br>D0-D42
Martin et al,<br>(43)<br>2008<br>USA	Safety and Immunogenicity,<br>Open-Label Phase 1 Trial<br>32 Weeks<br>N=10<br>21-49 y/o	SARS-CoV-1	DNA<br>Plasmid	Full-Length SARS-CoV-1 Spike GP	Experimental = 4mg (N=10)<br>Boost: D28, D56<br>IM	**ELISA-IgG**<br>D0-D224<br>**PRNT**<br>D0-D224<br>**Lentiviral Pseudotyped nAb Assay**<br>D0-D224	**IFNγ Linked ELISpot**<br>D0-D224<br>**Flow cytometry/ICS**<br>D0-D224
Lin et al,<br>(44)<br>2007<br>China	Safety and Immunogenicity,<br>Randomized, Double-Blind,<br>Placebo-Controlled Phase 1 Trial<br>30 Weeks<br>N=36<br>21-40 y/o	SARS-CoV-1	Inactivated Virus<br>ß-Propiolactone	SARS-CoV-1 Virus	Low Dose - 16 SU (N=12)<br>High Dose - 32 SU (N=12)<br>Control = Saline/Alum (N=12)<br>Boost: Day 28<br>IM	**ELISA-IgG**<br>D0-D56<br>**Live nAb Assay**<br>D0-D56	ND
Modjarrad et al,<br>(45)<br>2019<br>USA	Safety and Immunogenicity,<br>Open-Label, Dose-Escalation,<br>Single-Arm Phase 1 trial<br>60 Weeks<br>N=75<br>18-55 y/o	MERS-CoV	DNA Plasmid<br>(GLS-5300)	Full-length MERS-CoV spike GP	Low Dose = 0.67mg (N=25)<br>Intermediate Dose = 2mg (N=25)<br>High Dose = 6mg (N=25)<br>Boost: D28, D84<br>IM	**ELISA-IgG**<br>D0-D420<br>**Live nAb Assay**<br>D0-D420	**IFNγ Linked ELISpot**<br>D0-D420<br>**Flow cytometry/ICS**<br>D0-D98

SARS-CoV-2: Severe acute respiratory syndrome coronavirus 2, MERS-CoV: Middle east respiratory syndrome, SARS-CoV-1: Severe acute respiratory syndrome coronavirus 1, Ab: Antibody, nAb: Neutralizing antibody, AdH5: Recombinant human adenovirus 5, GP: Glycoprotein Vp: Viral particle, IM: Intramuscular, Enzyme-linked immunosorbent assay, IgG/A: Immunoglobulin G/A, IFNγ Interferon gamma, ELISpot: Enzyme-linked immunospot, ICS: Intracellular staining, RT-PCR: Reverse transcription polymerase chain reaction, ChAdOx1: Replication-deficient simian adenovirus vaccine vector oxford 1, MenACWY: Meningococcal vaccine, MNA: Microneutralization assay, IC: Inhibitory concentration, VSV: Vesicular stomatitis virus, mRNA: Messenger ribonucleic acid, LNP: Lipid nanoparticle, S2-P: SARS-CoV-2 GP with a transmembrane anchor and intact S1-S2 cleavage site, ug: Microgram, PRNT: Plaque reduction neutralization assay, MVA: Recombinant Modified Vaccinia virus Ankara, PFU: Plaque forming units, mg: Milligrams, DNA: Deoxyribonucleic acid, HEK293T: Human embryonic kidney 293 cells, SU, SARS-CoV-1 units,, ND: Not defined

Supplementary Table 4: Characteristics of the NHP studies

Study ID	Study Outcomes, Duration, Population (N)	NHP Species Age (Years)	Pathogen	Vaccine Platform	Antigen Insert	Route	Vaccine Groups, Dose, Dose Intervals, Vaccine groups, Boost/No Boost	Measure of Ab Response	Measure of Cellular Response	Measure of Efficacy Strain, Dose
Mercado et al., (46) 2020 USA	Immunogenicity, Safety (ADE), Protective Efficacy, 8 Weeks, N=52	Rhesus Macaques, 6-12	SARS-CoV-2	AdH26	SARS-CoV-2 Spike GP variants	IM	Immunogenicity<br>tPA.S - (n=4)<br>tPA.S.PP - (n=4)<br>tPA.WT.S - (n=4)<br>S - (n=4)<br>S.dCT - (n=4)<br>S.dTM.PP - (n=6)<br>S.PP - (n=6)<br>Sham - (20)<br>1x10^{6}vp<br>Not Boosted	ELISA-IgG, Live virus nAb assay, Lentivirus pseudotyped assay, PFU assay	IL-4/IFNγ ELISpot Flow cytometry/ICS	RT-PCR, HEK293T pseudovirus nAB assay,
Van Doremalen et al (47) 2020 UK	Immunogenicity, Protective Efficacy, Safety, 9 Weeks N=18	Rhesus Macaques 2-4yo	SARS-CoV-2	ChAdOx	SARS-CoV-2 Spike GP with tPA optimization	IM	Immunogenicity<br>Prime – (n=6)<br>Prime-Boost – (n=6)<br>Control – (n=6)<br>2.5x10^{10}vp<br>0 and 4th week<br>Boosted	ELISA-IgG/IgM, Live virus nAb Assay,	IFNγ ELISpot Flow cytometry/ICS	RT-PCR, Histopathology, Clinical observation, SARS-CoV-2 strain nCoV-WA1-2020, 4x10^{5}TCID$_{50}$
Corbett et al., (48) 2020 USA	Immunogenicity, Protective efficacy, 9 Weeks, N=24	Rhesus Macaques, 3-6y/o	SARS-CoV-2	DNA mRNA-1273 (LNP)	SARS-CoV-2 Spike S2-P	IM	Immunogenicity<br>Low dose - 10ug (n=8)<br>High dose - 100ug (n=8)<br>Control - PBS (n=8)<br>Week 0,4<br>Boosted	ELISA-IgG, Lentivirus pseudotyped assay, Live virus nAb assay	Flow cytometry/ICS	RT-PCR,, Histopathology, SARS-CoV-2-USA-WA1/2020, 7.6x10^{5}PFU
Wang et al (49) 2020 China	Safety evaluation. Immunogenicity, Protective Efficacy, 5 weeks N=40	Rhesus Macaques 3-6y/o	SARS-CoV-2	Inactivated vaccine + Alum BBIBP-CorV	Inactivated SARS-CoV-2	IM/IP	Immunogenicity<br>Group 1 – 2ug (n=10)<br>Group 2 – 4ug (n=10)<br>Group 3 – 8ug (n=10)<br>Boosted D7-D14<br>Challenge<br>Group 1 – 2ug (n=4)<br>Group 2 – 8ug (n=4)<br>Group 3 – Placebo (n=2)<br>Boosted D14	Live nAb assay	NA	RT-PCR, Histology, Clinical evaluation, Biochemical analysis 10^{6}TCID$_{50}$
Gao et al, (50) 2020 China	Immunogenicity, Safety (ADE), Protective Efficacy, 4 Weeks, N=40	Rhesus Macaques, 3-4	SARS-CoV-2	Inactivated Virus By β-Propiolactone	Inactivated SARS-CoV-2	IM	Immunogenicity/Vaccine Safety<br>High Dose - 6ug (n=10)<br>Low Dose - 1.5ug (n=10)<br>Sham (n=10)<br>Placebo (n=10)<br>0, 1, 2 Week<br>Challenge<br>High Dose - 6ug (N=4)<br>Medium Dose - 3ug (N=4)<br>Sham - (N=4)<br>Control - (N=4)<br>0, 1, 2 Week<br>Boosted	ELISA IgG, Live virus nAb assay	ND	RT-PCR, ELISA IgG, Live virus nAB assay, Histopathology, Biochemical analysis, Flow cytometry/ICS, Clinical observation, SARS-CoV-2-CN1 1x10^{6}TCID$_{50}$
Guebre-Xabier et al (51) 2020 USA	Immunogenicity, Protective Efficacy, 7 weeks N=12	Cynomolgus Macaques >3y/o	SARS-CoV-2	Recombinant Subunit and Matrix M Adjuvant NVX-CoV2373	SARS-CoV-2 Spike GP with perfusion stabilization	IM	Group 1 – 5ug + 50ug adjuvant (n=4)<br>Group 2 - 25ug + 50ug adjuvant (n=4)<br>Group 3 – 2.5ug + 25ug adjuvant (n=4)<br>Boosted D21	ELISA-IgG Cytopathic effect assay (CPE)	NA	RT-PCR, Histology SARS-CoV-2 strain nCoV-WA1-2020, 1.1x10^{4}PFU
Yang et al (52) 2020 USA	Immunogenicity, Protective efficacy 9 weeks N=32	Rhesus Macaques 5-9y/o	SARS-CoV-2	Recombinant subunit	SARS-CoV-2 RBD	IM	Immunogenicity<br>Experimental - 40ug + Alum (n=10)<br>Control – PBS + Alum (n=10)<br>Boosted D7<br>Challenge<br>Experimental – 40ug + Alum (n=4)<br>Experimental – 20ug + Alum (n=3)<br>Alum - (n=2)<br>Control – PBS (n=3)	ELISA-IgG, VSV-pseudovirus nAb assay	NA	RT-PCR, ND, !0^{6}PFU

Wang et al,<br>(53)<br>2015<br>USA	Immunogenicity,<br>18 Weeks,<br>N=18	Rhesus<br>Macaques,<br>4.4y/o	MERS-CoV	DNA plasmid	MERS-CoV full-length Spike GP	IM	Immunogenicity<br>DNA Group -1mg (n=6)<br>Week 0, 4, 8<br>DNA-S1 Group -1mg (n=6)<br>Week 0, 4, 8<br>Protein only group - 100mg S1 (n=6)<br>Week 0, 8<br>Challenge<br>Group 1 - Unvaccinated (n=6)<br>Group 2 - S-DNA/S1 Vaccinated (n=6)<br>Group 3 -S1/S1 Vaccinated (n=6)<br>Boosted	ELISA-IgG,<br>PRNT,<br>Huh7.5<br>Pseudovirus<br>nAb assay	ND	Clinical Observation,<br>Radiology,<br>JordanN3 MERS-CoV,<br>$5x10^6$vp
Yu et al,<br>(54)<br>2020<br>USA	Immunogenicity,<br>Protective Efficacy,<br>Safety (ADE),<br>CoP Investigation,<br>6 Weeks,<br>N=35	Rhesus<br>Macaques,<br>6-12	SARS-CoV-2	DNA Plasmid	SARS-CoV-2<br>Spike GP<br>Variants	IM	Immunogenicity<br>S - (n=4)<br>S.dCT - (n=4)<br>S.dTM - (n=4)<br>S1 - (n=4)<br>RBD - (n=4)<br>S.dTM.PP - (n=4)<br>Sham Control - (n=10)<br>5mg<br>0 and 3rd week<br>Boosted	ELISA IgG,<br>Live virus nAb<br>Assay,<br>HEK293T<br>pseudovirus<br>nAB assay	IFNγ ELISpot<br>Flow<br>cytometry/ICS	RT-PCR,<br>ELISA IgG,<br>Live virus nAB Assay,<br>HEK293T pseudovirus<br>nAB assay,<br>Flow cytometry/ICS,<br>Clinical observation,<br>ND,<br>$1.2x10^8$vp
Erasmus et al,<br>(55)<br>2020<br>USA	Immunogenicity,<br>Safety,<br>14 Weeks,<br>N=5	Pigtail<br>Macaques,<br>3-6y/o	SARS-CoV-2	RNA<br>LION/repRNA-CoV2S	SARS-CoV-2<br>Spike GP	IM	Immunogenicity<br>Group 1 - 250ug (n=3)<br>Group 2 - 50ug (n=2)<br>Group 1 Not Boosted<br>Group 2 Boosted<br>Week 4	ELISA-IgG,<br>MLV<br>pseudotyped<br>assay,<br>Vero cell<br>Pseudovirus<br>nAb assay	IFNγ ELISpot<br>Flow<br>cytometry/ICS	ND
Muthamani et al,<br>(56)<br>2015<br>USA	Immunogenicity,<br>Protective Efficacy,<br>12 weeks,<br>N=12	Rhesus<br>Macaques,<br>ND	MERS-CoV	DNA Plasmid	MERS-CoV full-length Spike GP	IM	Immunogenicity<br>Low dose group - 0.5mg (n=3)<br>High dose group - 2mg (n=3)<br>Control group - 2mg (Empty vector) (n=3)<br>Week 0, 3, 7<br>Boosted	ELISA-IgG,<br>HEK293T<br>Pseudovirus<br>nAB Assay,<br>Live Virus nAb<br>Assay	IFNγ ELISpot	RT-PCR,<br>Radiological,<br>Histopathology,<br>Clinical Evaluation,<br>EMC-2012-MERS-CoV,<br>$7x10^6TCID_{50}$
Kobinger et al,<br>(57)<br>2007<br>Canada	Immunogenicity,<br>38 Weeks,<br>N=4	Rhesus<br>Macaques,<br>Adult	SARS-CoV-1	AdH5/AdC7	SARS-CoV-1<br>Spike ORF	IM	Immunogenicity<br>Experimental - (n=4)<br>$1x10^{10}$vp AdH5 (1st injection)<br>$1x10^{12}$vp AdC7 (2nd injection)<br>Week 0, 13<br>Boosted	Live virus nAb<br>assay	IFNγ ELISpot<br>Flow<br>cytometry/ICS	ND
Qin et al,<br>(58)<br>2006<br>China	Immunogenicity,<br>Safety (ADE),<br>Protective efficacy,<br>14 Weeks,<br>N=29	Cynomolgus<br>Macaques,<br>4-5y/o	SARS-CoV-1	Inactivated<br>Vero Cell<br>With β-Propiolactone	Inactivated<br>SARS-CoV-1<br>virion	IM	Immunogenicity<br>Group 1 - Purified Vaccine + Alum 15ug (n=5)<br>Group 2 - Purified Vaccine 15ug (n=5)<br>Group 3 - Unpurified Vaccine 15ug (n=5)<br>Group 4 - Control 15ug (n=5)<br>Week 0, 1 , 3, 7<br>Safety<br>Group 1 - Purified inactivated 0.5ug (n=3)<br>Group 2 - Purified inactivated 1ug (n=3)<br>Group 3 - Purified inactivated 2ug (n=3)<br>Week 0, 1<br>Challenge<br>Group 2 - (n=4)<br>Control - (n=4)<br>Boosted	PRNT	ND	RT-PCR,<br>PRNT,<br>Histopathology,<br>Biochemical analysis,<br>Clinical observations<br>**Safety**<br>BJ-01 SARS-CoV-1,<br>$7x10TCID_{50}$<br>**Challenge**<br>GZ-01 SARS-CoV-1,<br>$7x10TCID_{50}$
Zhou et al,<br>(59)<br>2005<br>China	Immunogenicity,<br>Protective Efficacy,<br>Safety,<br>7 Weeks,<br>N=18	Rhesus<br>Macaques,<br>2-5y/o	SARS-CoV-1	Inactivated<br>Virus with<br>formaldehyde	Inactivated<br>SARS-CoV-1<br>virion	IM	Immunogenicity<br>Group 1 - 0.5ug (n=4)<br>Group 2 - 5ug (n=4)<br>Group 3 - 50ug (n=4)<br>Group 4 - 5000ug (n=2)<br>Group 5 – PBS (n=2)<br>Group 6 – PBS (n=2)<br>Week 0, 2<br>Boosted	ELISA-IgG/IgA,<br>Live virus nAb<br>Assay	ELISA-IFNγ/IL-4,<br>Flow<br>cytometry/ICS	RT-PCR,<br>ELISA-IgG,<br>Clinical Observation,<br>Histopathology,<br>Biochemical analysis,<br>Radiology,<br>NS-1 SARS-CoV-1,<br>$1x10^8$PFU
Ren et al<br>(60)<br>2020<br>China	Immunogenicity<br>5 Weeks<br>N=4	Rhesus<br>Macaques<br>3-4y/o	SARS-CoV-2	CHO-expressed recombinant subunit vaccine and CFA,<br>AD11.10 and AD20GOLD adjuvants	SARS-CoV-2 S1-Fc fusion protein	IM	Immunogenicity<br>Experimental – (N=4)<br>Boost D4-D9-D22-D26	ELISA-IgG,<br>HEK293T<br>pseudovirus<br>nAb assay,<br>Microdose<br>cytopathogenic<br>efficiency assay	NA	NA

Zhang et al<br>(61)<br>2020<br>China	Immunogenicity,<br>4 weeks<br>N=30	Cynomolgus<br>Macaques<br>Adult<br>Male/Female<br>3-6y/o	SARS-CoV-2	mRNA-LNP<br>ARCoV	SARS-CoV-2<br>RBD	IM	Group 1 – 100ug (n=10)<br>Group 2 - 1000ug (n=10)<br>Group 3 – Placebo (n=10)<br>Boosted D14	ELISA-IgG,<br>Huh7.5<br>Pseudovirus<br>nAb assay,<br>PRNT	IFNγ ELISpot<br>Flow<br>cytometry/ICS	NA
Lan et al,<br>(62)<br>2015<br>China	Immunogenicity,<br>Protective efficacy,<br>27 Weeks,<br>N=9	Rhesus<br>Macaques,<br>ND	MERS-CoV	Subunit	MERS-CoV<br>Spike GP: RBD	IM	<u>Immunogenicity</u><br>Low Dose = Prime - 50ug, Boost 1/2 – 100ug<br>rRBD admix + 1mg Alum (n=3)<br>High Dose = Prime - 200ug, Boost1/2 – 25ug<br>rRBD admix + 1mg Alum (n=3)<br>Mock Control = PBS (n=3)<br>Week 0, 8, 25<br>Boosted	ELISA-IgG,<br>Vero cell<br>Pseudovirus<br>nAb assay	IFNγ ELISpot	RT-PCR,<br>Live nAB assay,<br>Histopathology,<br>Radiology,<br>EMC-MERS-CoV,<br>6.5x10^{7}TCID$_{50}$
Sanchez-Felipe et al<br>(63)<br>2020<br>Belgium	Immunogenicity,<br>Protective Efficacy,<br>7 weeks<br>N=12	Cynomolgus<br>Macaques<br>Male	SARS-CoV-2	Yellow fever<br>17D vector	SARS-CoV-2<br>Spike GP	IM	Experimental (n=6)<br>Sham Control (n=6)<br>10^{6}PFU<br>Boosted D7	IgG Indirect<br>Immunofluores<br>cence assay,<br>HEK293T<br>pseudovirus<br>nAB assay,<br>PRNT	IFNγ ELISpot<br>Flow<br>cytometry/ICS	RT-PCR, Radiology,<br>Histology,<br>ND,<br>1.5x10^{4}TCID$_{50}$
Bukreyev et al,<br>(64)<br>2004<br>USA	Immunogenicity,<br>Protective Efficacy,<br>8 Weeks,<br>N=8	African Green<br>Monkeys,<br>ND	SARS-CoV-1	Attenuated<br>Parainfluenza<br>Virus	SARS-CoV-1<br>full-length<br>Spike GP	IN/IT	<u>Immunogenicity</u><br>Single Dose - (n=4)<br>Control - (n=4)<br>Week 0<br>Not Boosted	Live virus nAb<br>assay	ND	RT-PCR,<br>Live Virus nAB assay,<br>ND,<br>1x10^{6}TCID$_{50}$
Feng et al<br>(65)<br>2020<br>China	Immunogenicity,<br>Protective efficacy<br>9 weeks<br>N=20	Rhesus<br>Macaques<br>Male/Female<br>6-14y/o	SARS-CoV-2	rAd-5<br>(Ad5-S-nb2)	SARS-CoV-2<br>Spike GP	IM/IN	<u>Immunogenicity</u><br>Group 1 - 1x10^{11}vp (n=4)<br>Group 2 - 5x10^{10}vp (n=4)<br>Group 3 - 1x10^{10}vp (n=4)<br>Group 4 – Control 1x10^{11}vp (n=2)<br><u>Challenge</u><br>Group 5: Non-vaccinated (n=6)	ELISA-IgG,<br>HEK293T<br>pseudovirus<br>nAb assay,<br>Lentivirus<br>pseudotyped<br>assay,<br>Microneutraliz<br>ation assay	IFNγ ELISpot	RT-PCR, Histology, PRNT,<br>nAB assay<br>ND<br>2x10^{4}TCID$_{50}$ or 400 TCID$_{50}$
Chen et al,<br>(66)<br>2005<br>USA	Immunogenicity,<br>Protective Efficacy,<br>7 Weeks,<br>N=8	Rhesus<br>Macaques,<br>ND	SARS-CoV-1	MVA	SARS-CoV-1<br>full-length<br>Spike GP	IM	<u>Immunogenicity</u><br>Experimental - (n=4)<br>1x10^{8}TCID$_{50}$ (1st Injection)<br>3x10^{8}TCID$_{50}$ (2nd Injection)<br>Control - (n=4)<br>Week 0,4,<br>Boosted	HEK293T<br>pseudovirus<br>nAb assay,<br>Antibody<br>Adsorption<br>assay	ND	RT-PCR,<br>Live Virus nAB assay,<br>PUMC01-SARS-CoV-1,<br>1x10^{5}TCID$_{50}$
Liu et al,<br>(67)<br>2018<br>China	Immunogenicity,<br>7 Weeks,<br>N=8	Rhesus<br>Macaques,<br>2y/o	MERS-CoV	rVSV	MERS-CoV full-<br>length Spike<br>GP	IM/IN<br>(Group 1)<br>IN<br>(Group 2)	<u>Immunogenicity</u><br>Group 1 - 2x10^{7} FFU (n=4)<br>Group 2 - 2x10^{7} FFU (n=4)<br>Week 0<br>Not Boosted	ELISA-IgG,<br>Live Virus nAb<br>Assay	IFNγ ELISpot	ND
Wang et al,<br>(68)<br>2017<br>China	Immunogenicity,<br>8 Weeks,<br>N=6	Rhesus<br>Macaques,<br>ND	MERS-CoV	Recombinant<br>Baculovirus	MERS-CoV<br>S, E, M<br>proteins	IM	<u>Immunogenicity</u><br>Group 1: 250ug VLP + 250ug Alum (n=3)<br>Control: PBS (n=3)<br>0,2,4,6 Weeks<br>Boosted	ELISA-IgG,<br>Live Virus nAb<br>Assay	IFNγ and IL-4<br>ELISpot	ND

SARS-CoV-2: Severe acute respiratory syndrome coronavirus 2, MERS-CoV: Middle east respiratory syndrome, SARS-CoV-1: Severe acute respiratory syndrome coronavirus 1, Ab: Antibody, nAb: Neutralising antibody, ADE: Antibody dependent enhancement, CoP: Correlate of protection, ab: Antibody, nAb: Neutralising antibody, GP: Glycoprotein, IM: Intramuscular, IN: Intranasal, DNA: Deoxyribonucleic acid Vp: Virus particle, TCID$_{50}$: 50% tissue culture infective dose, S: Full length spike protein, S.dCT: Spike protein with a deletion of cytoplasmic tail, S.dTM: Spike protein with a deletion of transmembrane domain and cytoplasmic tail reflecting the soluble ectodomain, S1: S1 domain with a foldon trimerization tag, RBD: Receptor binding domain, S.dTM.PP: soluble ectodomain with a deleted furin cleavage site, with proline mutations and a foldon trimerization tag, ELISA: Enzyme-linked immunosorbent assay, IgG/A: Immunoglobulin G/A, IFNγ: Interferon gamma, ELISpot: Enzyme-linked immunospot, ICS: Intracellular staining, RT-PCR: Reverse transcription polymerase chain reaction, ChAdOx1: Replication-deficient simian adenovirus vaccine vector oxford 1, HEK293T: Human embryonic kidney 293 cells, AdH26:Recombinant human adenovirus-26, S.PP: Wild type leader sequence with full-length S GP with a mutation of the furin cleavage site and proline stabilizing mutations, tPA.S: Leader sequence with full length S, tPA.S.PP: Tissue plasminogen with full-length S with mutations of the furin cleavage site and two proline stabilization mutations, tPA.WT.S: Tandem tPA and wild type leader sequences with full-length S, sgRNA: Subgenomic ribonucleic acid, MLV: Murine Leukaemia virus, ND: Not defined, LNP: Lipid nanoparticle, S2-P: SARS-CoV-2 GP with a transmembrane anchor and intact S1-S2 cleavage site, PBS: Phosphate-buffered saline, AdH5: Human adenovirus vector 5, AdC7: Chimpanzee adenovirus 7, ORF: Open reading frame, PRNT: Plaque reduction neutralisation assay, ug: Microgram, IL-4: Interleukin 4, MVA: Recombinant modified Vaccinia virus Ankara, IT: Intratracheal, rVSV: Recombinant vesicular stomatitis virus, FFU: Focus forming units, E: Envelope protein, M: Membrane protein , VLP: Virus-Like Particle, RBD: Receptor binding domain, S1: Spike GP domain 1, Huh-7.5: hepatocyte derived cellular carcinoma cell line.

Supplementary Table 5. Summary of vaccine studies in NHP

Candidate and Pathogen	Vaccine Platform	Description	Human	NHP
rRBD-SARS-CoV-2<br>SARS-CoV-2<br>(52)	Recombinant subunit	Recombinant receptor binding domain protein vaccine		☑
Unnamed DNA plasmid vaccine<br>MERS-CoV<br>(53)	DNA Plasmid	DNA plasmid expressing full-length spike or truncated S1 protein genes		☑
Unnamed DNA Vaccine<br>SARS-CoV-2<br>(54)	DNA	DNA vaccine encoding multiple optimized variations of the spike protein gene		☑
repRNA-CoV2S<br>SARS-CoV-2<br>(55)	RNA	Alphavirus derived replicating RNA vaccine expressing full-length spike protein gene in an inorganic lipid nanoparticle emulsion		☑
Unnamed DNA plasmid vaccine<br>MERS-CoV<br>(56)	DNA Plasmid	DNA plasmid expressing full-length consensus spike protein		☑
Unnamed adenovirus vaccine<br>SARS-CoV-1<br>(57)	AdH5/AdC7	Human Adenovirus 5 and Chimpanzee adenovirus 7 vaccine regimen expressing spike protein		☑
Unnamed inactivated virus vaccine<br>SARS-CoV-1<br>(58)	Inactivated Virus (BJ-01 strain)	β-propiolactone Inactivated vaccine		☑
Unnamed inactivated virus vaccine<br>SARS-CoV-1<br>(59)	Inactivated Virus (NS-1 strain)	Formaldehyde inactivated vaccine		☑
S1-Fc fusion<br>SARS-CoV-2<br>(60)	Recombinant Protein	CHO-expressed S1-Fc fusion protein with CFA, AD11.10 and AD20GOLD adjuvants		☑
ARCoV<br>SARS-CoV-2<br>(61)	mRNA	mRNA vaccine encoding the RBD, encapsulated in a lipid nanoparticle.		☑
rRBD-MERS-CoV<br>MERS-CoV<br>(62)	Recombinant subunit	Recombinant receptor binding domain protein vaccine administered with Alum adjuvant		☑
YF-S<br>SARS-CoV-2<br>(63)	Live-attenuated yellow fever virus	Yellow fever 17D: Single stranded, live attenuated virus vaccine		☑
Unnamed live-attenuated vaccine<br>SARS-CoV-1<br>(64)	Live attenuated Parainfluenza virus	Attenuated Parainfluenza virus vaccine expressing full length spike protein		☑
Ad5-S-nb2<br>SARS-CoV-2<br>(65)	AdH5	Replication deficient adenovirus 5 vectored vaccine expressing codon optimized full-length spike protein.		☑
Unnamed live-attenuated vaccine<br>SARS-CoV-1<br>(66)	rMVA	Live-attenuated Modified Vaccina Ankara (Poxvirus) expressing full-length spike protein		☑
VSVΔG-MERS<br>MERS-CoV<br>(67)	VSV	Vesicular stomatitis virus encoding the MERS-CoV S gene		☑
MERS-CoV-VLP<br>(68)	VLP	Recombinant Baculovirus expressing the spike, envelope and membrane genes		☑

Supplementary Table 6: Demographic characteristics of the human studies

						**Race/Ethnic group**						
	**n**	**Age (Mean)**	**Male (n)**	**Female (n)**	**BMI Kg/m²**	**White**	**Hispanic**	**Black/African American**	**Asian**	**Multiracial**	**Other**	**Native Hawaiian/ Pacific Islander**
Zhu rAdH5, (1) (14)	108	36.3	55	53	23.7	-	-	-	-		-	
Zhu rAdH5, (2) (15)	508	39.6	254	254	23.9	-	-	-	-	-	-	-
Folegatti, ChAdOx (1) (23)	24	25.8	7	17	-	24	-	-	-	-	-	-
Modjarrad, DNA (45)	75	32.2	44	31	-	37	4	31	6	-	1	-
Koch, MVA (42)	26	31.5	8	24	23.3	27	2	1	1	-	-	-
Martin, DNA (43)	10	35.5	7	3	24.6	-	2	-	-	-	8	-
Folegatti, ChAdOx (2) (19)	1077	35	541	536	24	979	-	6	44	-	48	-
Mulligan, BNT162b1 (29)	45	35.4	23	22	-	37	2	1	7	-	-	-
Xia, Inactivated (38)	320	42.8	120	200	-	-	-	-	-	-	-	-
Keech, NVX-CoV2373 (39)	131	30.8	66	65	25.1	103	19	2	17	1	7	1
Logunov, rAdH26/5 (16)	76	27.4	53	23	-	74	-	-	2	-	-	-
Che, Inactivated (32)	742	39.2	283	467	23.9	-	-	-	-	-	-	-
Walsh, BNT162b1/2 (28)	195	52.4	47	58	-	87	1	1	2	-	-	-
Sahin, BNT162b1 (30)	60	37.5	30	30	-	58	-	1	1	-	-	-
Anderson, mRNA-1273 (25)	40	68.7	19	21	25	39	1	-	1	-	-	-
Xia, BBIBP-CorV (33)	640	47.7	293	347	-	-	-	-	-	-	-	-
Zhang, CoronaVac (34)	744	42.4	346	397	-	-	-	-	-	-	-	-

Polack, BNT162b2 (31)	43,448	52	19075	18631	-	31266	10543	3492	1608	855	409	76
Ramasamy, ChAdOx (20)	560	61.7	277	275	24.5	524	-	1	19	4	4	-
Jackson, mRNA-1273 (24)	45	33	22	23	25.3	40	6	2	1	-	2	1
Baden, mRNA-1273 (27)	30,420	51.4	15985	14366	29.3	24024	6235	3090	1382	636	637	67
Voysey, ChAdOx (21)	23,848	NA	10485	13260	24.8	17864	-	2394	825	2452	210	-
Sadoff, Ad26.COV2.S (18)	805	52.6	391	213	25	761	23	23	10	1	9	1
Tebas, DNA-INO-4800 (40)	40	35.3	22	18	-	33	0	2	5	-	-	-
Ella, BBV152 I (36)	375	NA	297	78	23.5	-	-	-	-	-	-	-
Logunov, rAdH26/5 III (17)	19866	45.3	12158	7708	26.75	19571	-	-	286	-	9	-
Wu, CoronaVac (35)	421	66.5	206	215	-	-	-	-	421	-	-	-
Chu, mRNA-1273 (26)	600	50.9	210	390	25.3	569	-	16	7	4	3	1
Richmond, SCB-2019 (41)	151	45.2	64	87	-	130	10	1	16	-	2	-
Ella, BBV152 II (37)	380	NA	285	95	25	-	-	-	-	-	-	-
Voysey, ChAdOx (22)	17178	NA	7482	9696	25.4	12975	-	1693	1210	1753	167	-
Total	142,958	42.7	50.6%	49.4%	24.9	72.78%	11.23%	7.17%	3.91%	3.80%	1.01%	0.10%

Zhu AdH5 (1): AdH5-SARS-CoV-2 phase 1 vaccine, Zhu AdH5 (2): AdH5-SARS-CoV-2 phase 2 vaccine, Folegatti ChAdOx (1): ChAdOx-MERS-CoV vaccine, Folegatti ChAdOx (2): ChAdOx-SARS-CoV-2 vaccine.

Supplementary Table 7: Vaccine efficacy against COVID-19

Vaccine Study	Number of study participants (n)			Efficacy End Point	Vaccine	Placebo	Vaccine efficacy (%) (95% CI)
					No of COVID-19 cases	No of COVID-19 cases	
Polack, BNT162b2 (31)	40137			COVID-19 infection 7 days after 2nd vaccination in those with and without symptoms of infection.	9	169	94.6% (89.9-97.3)
Baden, mRNA-1273 (27)	30352			Covid-19 Infection 14 days after the second injection with at least two of the following symptoms: fever (temperature ≥38°C), chills, myalgia, headache, sore throat, or new olfactory or taste disorder	11	185	94.1 (89.3-96.8)
Voysey, ChAdOx (21)	11636			NAAT confirmed COVID-19 with a qualifying symptom (Fever, cough, anosmia, shortness of breath and ageusia)	30	101	70.4% (54.8-80.6)
Logunov, rAd26/5 (17)	19866			Covid-19 Infection 21 days after first injection.	16	62	91.6% (85.6-95.2)
Voysey, ChAdOx (22)	17178	Total Efficacy		Primary symptomatic Covid19 more than 14 days after first dose.	84/8597	248/8581	66.7% (57.4-74.0)
		Standard Dose/standard Dose	<6 weeks		35/3890	76/3856	55.1% (33.0-69.9)
			6-8 weeks		20/1112	44/1009	59.9% (32.0-76.4)
			9-11 weeks		11/906	32/958	63.7% (28.0-81.7)
			≥12 weeks		8/1293	45/1356	81.3% (60.3-91.2)
		Low Dose/Standard dose			10/1396	51/1402	80.7% (62.1–90.2)
		Single Standard dose		Primary symptomatic Covid19, 22-90 days after dose.	17/9257	71/9237	76.0% (59.3-85.9)
		Standard Dose/standard Dose	<6 weeks	Asymptomatic COVID-19 cases more than 14 days after second dose	9/728	8/733	−11.8% (−189.5-56.8)
			6-8 weeks		14/528	7/476	−74.2% (−330.3-29.5)
			9-11 weeks		6/599	11/666	39.9% (−62.3-77.8)
			≥12 weeks		12/837	16/876	22.8% (−63.3-63.5)
		Standard Dose/standard Dose	<6 weeks	Any NAAT-positive COVID-19 cases more than 14 days after second dose	51/3890	94/3856	47.1% (25.6-62.4)
			6-8 weeks		39/1112	51/1009	32.6% (−2.2- 55.5)
			9-11 weeks		18/906	50/958	61.9% (34.8-77.8)
			≥12 weeks		24/1293	63/1356	59.9% (35.8-75.0)

Supplementary Table 8: NHP safety analysis

First author, Vaccine platform, Pathogen	Type of analysis	Summary
Zhou, 2005 (59)<br>Inactivated virus,<br>SARS-CoV-1	Clinical signs, biochemical and hematological parameters	• No fluctuations in hematological or biochemical parameters<br>• High dose vaccine caused mild induration in one subject
Qin, 2006 (58)<br>Inactivated virus,<br>SARS-CoV-1	ADE, Clinical signs, biochemical and haematological parameters	• No evidence of ADE<br>• No change in biochemical or haematological measures
Gao, 2020 (50)<br>Inactivated virus,<br>SARS-CoV-2	ADE/Th2, Clinical signs, hematological and biochemical parameters.<br>Histopathology	• No evidence of ADE even in the presence low nAB titer in some vaccine groups<br>• No Th2 pathology registered<br>• No change in biochemical, histopathological, or hematological parameters
Erasmus, 2020 (55)<br>RNA,<br>SARS-CoV-2	Clinical signs, biochemical and hematological parameters	• No fluctuations in biochemical, hematological or biochemical parameters
Yu, 2020 (54)<br>DNA,<br>SARS-CoV-2	ADE	• No Th2 skewing and no evidence of ADE
Corbett, 2020 (48)<br>mRNA-1273,<br>SARS-CoV-2	Histopathology<br>Lung inflammation assessment<br>Th2 responses	• Inflammation mild in low dose group<br>• No Inflammation in high dose group<br>• No vaccine associated lung immunopathology<br>• No Th2 responses
Wang, 2020 (49)<br>BBIBP-CorV<br>SARS-CoV-2	ADE, Clinical signs, biochemical and Hematological parameters	• Local injection site inflammation in all groups observed at D25<br>• No significant clinical abnormalities<br>• No change in pathological indicators or lymphocyte subsets

Supplementary Table 9. Interstudy analysis of individual local adverse events reported in vaccine group (excluding control group) of coronavirus vaccine studies. Data represent % of patients in experimental group reporting any grade of symptomatic adverse event.

Study (% Patients)	Injection site pain	Redness	Swelling	Induration	Pruritus	Tenderness	Warmth	Bruising
Zhu, AdH5 (Phase 1) (n=108)	54.0	4.0	7.0	4.0	5.0	-	-	-
Zhu, AdH5 (Phase 2) Low Dose (n=129)	56.0	1.0	4.0	2.0	4.0	-	-	-
Zhu, AdH5 (Phase 2) High Dose (n=253)	57.0	2.0	4.0	10.0	5.0	-	-	-
Logunov AdH26/5-Vac (n=38)	55.3	-	2.6	-	2.6	-	5.3	-
Logunov AdH26/5-Lyo (n=38)	63	-	0.0	-	0	-	2.6	-
Sadoff, Ad26.COV2.S (n=805)	55.0	2.5	2.8	-	-	-	-	-
Ramasamy ChAdOx Prime (n=128)	29.6	1.9	0.9	0.2	5.0	53.1	4.9	-
Ramasamy ChAdOx Boost (n=128)	19.9	2.3	1.5	0.4	5.0	44.0	5.9	-
Folegatti, ChAdOx (Phase 1/2) (n=543)	65.6	2.9	4.1	3.1	7.0	82.1	24.5	-
Baden, mRNA-1273 Prime (n=15,210)	83.7	2.8	6.1	-	-	10.2	-	-
Baden, mRNA-1273 Boost (n=15,210)	88.2	8.6	12.2	-	-	14.2	-	-
Jackson mRNA-1273 Prime (n=45)	86.7	6.7	10.7	-	-	-	-	-
Jackson mRNA-1273 Boost (n=45)	91.7	11.0	9.7	-	-	-	-	-
Anderson mRNA-1273 Prime (n=40)	62.5	0.0	-	2.5	-	-	-	-
Anderson mRNA-1273 Boost (n=40)	75.0	17.5	-	20.0	-	-	-	-
Chu mRNA-1273 Prime (n=600)	74.5	3	4	-	-	-	-	-
Chu mRNA-1273 Boost (n=600)	82.5	6.5	8.5	-	-	-	-	-
Polack, BNT162b2 Prime (n=21,720)	77.0	5.0	6.5	-	-	-	-	-
Polack, BNT162b2 Boost (n=21,720)	72.0	6.5	6.5	-	-	-	-	-
Mulligan BNT162b1 Prime (n=36)	86.0	15.0	18.3	-	-	-	-	-
Mulligan BNT162b1 Boost (n=24)	90.0	7.5	11.0	-	-	-	-	-
Walsh BNT162b1 Prime (n=84)	79.2	2.8	12.5	-	-	-	-	-
Walsh BNT162b1 Boost (n=72)	82.0	5.5	13.8	-	-	-	-	-
Walsh BNT162b2 Prime (n=72)	65.3	1.3	2.8	-	-	-	-	-
Walsh BNT162b2 Boost (n=72)	63.7	0.0	0.0	-	-	-	-	-
Che inactivated SARS-COV-2 (n=600)	14.0	0.5	0.0	-	2.0	-	-	-
Xia BBIPB Phase I (n=144)	24.0	0.5	0.5	2.5	1.0	-	-	-
Xia BBIPB Phase II (n=336)	16.0	1.0	2.0		1.0	-	-	-
Zhang CoronaVac (n=570)	16.1	1.0	0.8	0.5	0.5	-	-	-
Wu, Coronavac I/II (n=421)	10.3	0.9	0.6	-	0.9	-	-	-
Ella, BBV152 Prime I (n=300)	3.7	-	0.0	-	-	-	-	-
Ella, BBV152 Boost I (n=300)	1.3	-	0.0	-	-	-	-	-
Ella, BBV152 II (n=380)	7	1	-	-	-	-	-	-
Keech NVX-CoV2373 Prime (n=102)	42.2	1.0	-	0.0	-	48.7	-	-
Keech NVX-CoV2373 Boost (n=76)	47.2	6.1	-	4.7	-	60.6	-	-
Tebas, INO-4800 Prime (n=40)	5.0	2.5	-	0.0	-	-	-	-
Tebas, INO-4800 Boost (n=40)	2.5	0.0	-	0.0	-	-	-	-
Richmond, SCB-2019 Prime (n=121)	28.3	3.3	3.3	-	-	-	-	-
Richmond, SCB-2019 Boost (n=121)	32.5	10.8	10.0	-	-	-	-	-
Koch MERS MVA (n=26)	65.0	42.0	38.0	38.0	-	-	-	27.0
Martin SARS DNA (n=10)	100.0	40.0	20.0	-	-	-	-	-
Lin SARS Inactivated (n=24)	29.2	8.4	-	-	4.2	-	-	-
Modjarrad GLS-5300 MERS (n=75)	92.0	6.7	-	2.7		84.0	-	1.3
Folegatti, ChAdOx 1 MERS (n=24)	80	20	0	-	21.0	-	20	-

Supplementary Table 10: Interstudy analysis of individual systematic adverse events reported in vaccine group (excluding control group) of coronavirus vaccine studies. Data represent % of patients in experimental group reporting any grade of symptomatic adverse event.

Study (% Patients)	Headache	Myalgia	Fatigue	Arthralgia	Fever	Nausea	Chills	Diarrhoea	Appetite impairment	Vomiting	Abdominal Pain	Hyper-sensitivity	Itch	Mucosal abnormality	Feverishness	Cough
Zhu, AdH5 (Phase 1) (n=108)	39	17	44	8	46	6	-	11	16	2	-	-	3	1	-	6
Zhu, AdH5 (Phase 2) Low Dose (n=129)	28	18	34	10	16	5	-	8	5	1	-	0	3	2	-	2
Zhu, AdH5 (Phase 2) High Dose (n=253)	29	15	42	13	32	8	-	8	11	2	-	0	2	1	-	5
Logunov, AdH26/5-Vac (n=38)	52.6	26.3	44.7	26.3	81.6	-	-	10.5	7.8	-	-	-	-	-	-	5.3
Logunov, AdH26/5-Lyo (n=38)	31.6	23.7	10.5	23.7	23.7	-	-	0	0	-	-	-	-	-	-	0
Sadoff, Ad26.COV2.S (n=805)	43.5	37	41.1	-	15.8	15.5	-	-	-	-	-	-	-	-	-	-
Ramasamy, ChAdOx Prime (n=128)	36.6	27.3	41.5	13.8	5.7	7.3	13.0	-	-	-	-	-	-	-	16.9	-
Ramasamy, ChAdOx Boost (n=128)	19.6	18.1	23.5	5.0	0.0	6.2	4.3	-	-	-	-	-	-	-	7.3	-
Folegatti, ChAdOx (Phase 1/2) (n=543)	67.2	59.1	70.0	30.6	17.7	26.3	52.9	-	-	-	-	-	-	-	49.7	-
Baden, mRNA-1273 Prime (n=15,210)	32.7	22.7	37.2	16.6	0.8	8.3	8.3	-	-	-	-	-	-	-	-	-
Baden, mRNA-1273 Boost (n=15,210)	58.6	58	65.3	42.8	15.5	19	44.2	-	-	-	-	-	-	-	-	-
Jackson, mRNA-1273 Prime (n=45)	31.0	13.7	29.3	6.7	0.0	5.0	6.7	-	-	-	-	-	-	-	-	-
Jackson, mRNA-1273 Boost (n=45)	60.7	56.3	62.7	27.7	32.0	24.0	59.3	-	-	-	-	-	-	-	-	-
Anderson, mRNA-1273 Prime (n=40)	12.5	20	25	10	0	0	-	-	-	-	-	-	-	-	-	-
Anderson, mRNA-1273 Boost (n=40)	40	50	50	12.5	7.5	17.5	-	-	-	-	-	-	-	-	-	-

**Chu, mRNA-1273 Prime (n=600)**	25.5	12.5	24.5	9	0.0025	4.5	6	-	-	-	-	-	-	-	-	-
**Chu, mRNA-1273 Boost (n=600)**	51	47.5	59	37	9.5	17	32	-	-	-	-	-	-	-	-	-
**Polack, BNT162b2 Prime (n=21,720)**	33.5	17.5	40.5	10	2.5	-	10	9.5	-	0.5	-	-	-	-	-	-
**Polack, BNT162b2 Boost (n=21,720)**	45.5	33	55	20.5	13.5	-	29	9	-	1.5	-	-	-	-	-	-
**Mulligan, BNT162b1 Prime (n=36)**	55	29.3	55.7	13.3	22.0	-	49.0	18.3	-	0	-	-	-	-	-	-
**Mulligan, BNT162b1 Boost (n=24)**	87.5	50	75	25	41.5	-	47.5	4	-	0	-	-	-	-	-	-
**Walsh, BNT162b1 Prime (n=84)**	46.4	28.5	45.8	16.6	6.8	-	20.7	10.6	-	0	-	-	-	-	-	-
**Walsh, BNT162b1 Boost (n=72)**	73.6	45.6	63.8	29.0	25.5	-	43.0	6.93	-	1.38	-	-	-	-	-	-
**Walsh, BNT162b2 Prime (n=72)**	24.9	13.9	29.2	4.1	2.8	-	8.3	2.8	-	1.38	-	-	-	-	-	-
**Walsh, BNT162b2 Boost (n=72)**	36.0	26.4	45.8	8.3	5.5	-	25.0	0	-	1.38	-	-	-	-	-	-
**Che, inactivated SARS-COV-2 (n=600)**	-	-	5.33	-	2.70	0.83	-	1.67	-	0	-	0.6	-	0.33	-	1.83
**Wu, Coronavac I/II (n=421)**	1.4	1.1	3.4	0.0	3.7	0.6	-	1.1	-	0.3	0.0	0.3	-	-	-	0.9
**Ella, BBV152 Prime I (n=300)**	2.7	1.0	2.0	-	1.7	1.7	-	-	-	-	-	-	-	-	-	-
**Ella, BBV152 Boost I (n=300)**	0.0	0.3	1.7	-	1.3	0.0	-	-	-	-	-	-	-	-	-	-
**Ella, BBV152 II (n=380)**	2.5	2	2.5	-	6	-	-	-	-	-	-	-	-	-	-	-
**Xia, BBIPB Phase I (n=144)**	2	0	2	0.5	3.5	-	-	1.5	0.5	1	-	-	1	1	-	-
**Xia, BBIPB Phase II (n=336)**	1	<1	3	-	2	1	-	-	-	-	-	-	1	-	-	1
**Zhang, CoronaVac (n=570)**	2.1	2.1	5.9	-	3.3	1.3	2.1	-	1.0	0.3	-	0.5	-	-	-	0.96
**Keech, NVX-CoV2373 Prime (n=102)**	32.3	23.3	29.6	8.9	0.0	9.9	-	-	-	-	-	-	-	-	-	-
**Keech, NVX-CoV2373 Boost (n=76)**	44.8	34.4	32.4	15.8	1.7	11.4	-	-	-	-	-	-	-	-	-	-
**Tebas, INO-4800 Prime (n=40)**	0	0	0	0	0	2.5	0	-	-	-	-	-	-	-	-	-
**Tebas, INO-4800 Boost (n=40)**	0	0	0	0	0	0	0	-	-	-	-	-	-	-	-	-

**Richmond, SCB-2019 Prime (n=121)**	16.7	10.8	10.8	-	0.0	1.7	-	2.5	-	2.5	-	-	-	-	-	-
**Richmond, SCB-2019 Boost (n=121)**	15.0	13.3	16.7	-	5.0	5.0	-	0.0	-	0.8	-	-	-	-	-	-
**Koch, MERS MVA (n=26)**	62	19	62	15	8	8	4	3	-	-	19	-	-	-	-	-
**Martin, SARS DNA (n=10)**	10	30	-	-	1	0	1	-	-	-	-	-	-	-	-	-
**Lin, SARS Inactivated (n=24)**	4.2	4.2	4.2	-	4.2	-	-	15.5	-	-	12.5	-	-	-	-	-
**Modjarrad, GLS-5300 MERS (n=75)**	30.7	14.7	18.7	5.3	-	12	-	-	-	-	-	-	-	-	-	-
**Folegatti, ChAdOx 1 MERS (n=24)**	66.7	54.2	66.7	33.3	20.8	33.3	-	-	-	-	-	-	-	-	50	-

Supplementary Table 11: Serious adverse events. Summary of events related to SARS-CoV-2 vaccine or placebo reported in humans studies. Vaccine study, population (n), number of serious adverse events and description of these events.

Vaccine Study	N	Description	Treatment Group
Baden, mRNA-1273 (27)	30420	(1) Autonomic nervous system imbalance<br>(2) Paraesthesia<br>(3) Dyspnoea<br>(4) Pulmonary embolism<br>(5) Nausea<br>(6) Vomiting<br>(7) Rheumatoid arthritis<br>(8) Polymyalgia rheumatica<br>(9) Swelling face<br>(10) Edema peripheral<br>(11) Feeling hot<br>(12) Immunization anxiety-related reaction<br>(13) Procedural haemorrhage	(1) Vaccine<br>(2) Placebo<br>(3) Vaccine<br>(4) Placebo<br>(5) Vaccine<br>(6) Vaccine<br>(7) Vaccine<br>(8) Placebo<br>(9) 1 Case Placebo, 2 Cases Vaccine<br>(10) Vaccine<br>(11) Placebo<br>(12) Placebo<br>(13) Placebo
Polack, BNT162b2 (31)	43488	(1) Shoulder injury related to vaccination<br>(2) Right axillary lymphadenopathy<br>(3) Paroxysmal ventricular arrhythmia<br>(4) Right leg paraesthesia	All vaccine
Voysey, ChAdOx (21)	23848	(1) Hemolytic anemia<br>(2) Transverse Myelitis<br>(3) Fever	(1) Placebo<br>(2) Vaccine<br>(3) Vaccine
Sadoff, Ad26.COV2.S (18)	805	(1) Pyrexia	(1) Vaccine
Folegatti, ChAdOx (19)	1077	(1) Haemolytic anaemia	(1) Placebo
Logunov, rAd26/5 (17)	21862	-	-

Supplementary Table 12: Pre-existing anti-vector neutralizing antibody titers recorded before vaccination and their correlation with anti-RBD seroconversion or SARS-CoV-2 neutralization in adenoviral vectored SARS-CoV-2 vaccines. n= number of patients with pre-existing neutralizing antibody titer available.

Vaccine group	n	No. participants with nAb titer = 0	No. participants with nAb titer 0<N≤200	No. participants with >200 nAb titer	Regression analysis: Vector nAb: Anti-RBD/SARS-CoV-2 nAb.	Summary
Zhu, AdH5 (Phase 1) Low dose (14)	36	NA	16(44%)	20(56%)	-1·67 (p=0·0003)	High levels of AdH5 nAbs at baseline. Significantly less nAb after vaccination in those with pre-existing AdH5 nAb.
Zhu, AdH5 (Phase 1) Middle dose (14)	36	NA	17(47%)	19(53%)		
Zhu, AdH5 (Phase 1) High dose (14)	36	NA	20(56%)	16(44%)		
Zhu, AdH5 (Phase 2) Low dose (15)	129	NA	54(42%)	75(58%)	-1·24 (p<0·0001)	High pre-existing AdH5 neutralizing antibody titer associated with reduced antibody responses, especially in those 55 years and older.
Zhu, AdH5 (Phase 2) High dose (15)	253	NA	127(50%)	126(50%)		
Folegatti, ChAdOx (Phase 2) (19)	98	79(81%)	18(18%)	1(1%)	No relationship	No association between pre-existing antibodies to the ChAdOx vector and antibody responses to SARS-CoV-2.
Logunov AdH26/5-Vac rAd26 (16)	38	32(84%)	3(8%)	3(8%)	0·07 (p=0·77)	No significant correlation between nAb titers to the adenoviral vector and the titer of RBD-specific IgG. No nAb cross reactivity between AdH5 and AdH26 adenoviral vectors.
Logunov AdH26/5-Vac rAd5 (16)	38	27(81%)	6(16%)	5(13%)	-0·2 (p=0·39)	
Logunov AdH26/5-Lyo rAd26 (16)	38	31(82%)	5(13%)	2(5%)	-0·44 (p=0·06)	
Logunov AdH26/5-Vac rAd5 (16)	38	29(76%)	5(13%)	4(11%)	-0·44 (p=0·06)	
Ramasamy ChAdOx (20)	552	522(100%)	0	0	NA	Anti-ChAdOx nAb titer increased following prime vaccination. No increase after boosting. Small negative correlation between Anti-ChAdOx nAb titre before boosting and anti-Spike IgG titer after boosting.
Sadoff rAd26.COV2.S (18)	303	303(99.4%)	2(0.6%)	0	-0.13 (NS)	No significant relationship between pre-existing neutralizing antibody titer and SARS-CoV-2 nAb induction. No relationship apparent after boost.

Supplementary Table 13. Human and NHP T cell analysis. *data from trial was estimated using graphical representations, **responses too low to extract.

<table>
<tr><th>Study Title
Vaccine Group</th><th>Pathogen,
Platform</th><th>Study
Subjects</th><th>Polyfunctionality</th><th>T cell subsets</th><th>Effector Function, Cytokine
Proportion (Mean, %)</th><th>Peptides</th><th>Summary</th></tr>
<tr><td>Ella, 2021 (36)</td><td>SARS-CoV-2
BBV152</td><td>Human</td><td>Yes – Week 7</td><td>• CD4+
IFNγ, IL-2, and TNFα (Th1)
IL-5, IL-10, and IL-13 (Th2)
Effector Memory
CD4+CD45RO+
CD4+CD45RO+CD27+</td><td>• NA</td><td>Overlapping Spike GP peptide pools covering the RBD protein</td><td>• Th1 bias observed on day 42
• Minimal Th2 responses observed in all vaccine groups
• Memory T cell response observed on day 104 in all vaccine and control groups</td></tr>
<tr><td>Richmond, 2021 (41)</td><td>SARS-CoV-2
S-Timer</td><td>Human</td><td>NA</td><td>• CD4
IFNγ+ and/or IL-2+ (Th1)
IL-4 and/or IL-5 (Th2)
IL-17 (Th17)</td><td>• NA</td><td>Spike GP peptide pools</td><td>• Th1 bias recorded in the adjuvanted vaccine groups
• No T cell responses in the non-adjuvanted group.
• Dose dependant increase in IFNγ and IL-2-positive CD4+ T-cells
• No Th2 or Th17 responses were observed in any vaccine group.</td></tr>
<tr><td>Zhu, 2020 (14)*
Phase 1 trial
Low Dose</td><td rowspan="3">SARS-CoV-2
AdH-5</td><td rowspan="3">Human</td><td rowspan="3">Yes – Week 2</td><td rowspan="3">• CD4+
IFNγ+, TNFα+, IL-2+
(Memory)
• CD8+
IFNγ+, TNFα+, IL-2+
(Memory)</td><td>• CD4
IFNγ+= 0.18%
TNFα+= 0.06%
IL-2+= 0.16%
• CD8
IFNγ+= 0.2%
TNFα+= 0.018%
IL-2+= 0.011%</td><td rowspan="3">Overlapping Spike GP peptide pools</td><td rowspan="3">• Higher polyfunctionality proportion in CD4+ memory T cells than CD8+ T cells
• IL-2 higher in CD4+ T cells than CD8+ T cells
• TNFα expression in CD4/CD8 lowest in low dose group (Highest = High dose)
• Higher polyfunctionality phenotypes associated with higher doses</td></tr>
<tr><td>Zhu, 2020 (14)*
Phase 1 trial
Inter Dose</td><td>• CD4
IFNγ+= 0.19%
TNFα+= 0.06%
IL-2+= 0.17%
• CD8
IFNγ+= 0.18%
TNFα+= 0.012%
IL-2+= 0.005%</td></tr>
<tr><td>Zhu, 2020 (14)*
Phase 1 trial
High Dose</td><td>• CD4
IFNγ+= 0.11%
TNFα+= 0.055%
IL-2+= 0.11%
• CD8
IFNγ+= 0.14%
TNFα+= 0.0125%
IL-2+= 0.004%</td></tr>
<tr><td>Anderson, 2020 (25)
Low Dose
56-70yo</td><td rowspan="2">SARS-CoV-2
mRNA-1273</td><td rowspan="2">Human</td><td rowspan="2">Yes – Week 6</td><td>• CD4+
IFNγ+, TNFα+, IL-2+, IL-4+, IL-13+
• CD8+
IFNγ+, TNFα+, IL-2+</td><td>• CD4
IFNγ+= 0.108%
TNFα+= 0.224%
IL-2+= 0.204%
IL-4+= 0.01%
IL-13+= 0.019%
• CD8
IFNγ+= 0.088%
TNFα+= 0.061%
IL-2+= 0.047%</td><td rowspan="2">Overlapping Spike GP peptide pools</td><td rowspan="2">• Strong CD4+ response in both 100ug groups and the 56-70yo 25ug group
• Effector function hierarchy: TNFα>IL-2>IFNγ
• Th2 response was minimal across all groups
• Lowest response was found in the 25ug >71y/o group
• CD8+ responses to S-2p were low after 2nd</td></tr>
<tr><td>Anderson, 2020 (25)</td><td>• CD4+
IFNγ+, TNFα+, IL-2+, IL-4+, IL-13+</td><td>• CD4
IFNγ+= 0.031%</td></tr>
</table>

**Low Dose**<br>**≥71yo**				• CD8+<br>IFNγ+, TNFα+, IL-2+	TNFα+= 0.085%<br>IL-2+= 0.065%<br>IL-4+= 0.005%<br>IL-13+=0.013%<br>• CD8<br>IFNγ+= 0.031%<br>TNFα+= 0.018%<br>IL-2+= 0.008%		vaccination in the 100ug groups
**Anderson, 2020**<br>**(25)**<br>**High Dose**<br>**56-70yo**				• CD4+<br>IFNγ+, TNFα+, IL-2+, IL-4+, IL-13+<br>• CD8+<br>IFNγ+, TNFα+, IL-2+	• CD4<br>IFNγ+= 0.148%<br>TNFα+= 0.296%<br>IL-2+= 0.224%<br>IL-4+= 0.021%<br>IL-13+= 0.018%<br>• CD8<br>IFNγ+= 0.058%<br>TNFα+= 0.036%<br>IL-2+= 0.029%		
**Anderson, 2020**<br>**(25)**<br>**High Dose**<br>**≥71yo**				• CD4+<br>IFNγ+, TNFα+, IL-2+, IL-4+, IL-13+<br>• CD8+<br>IFNγ+, TNFα+, IL-2+	• CD4<br>IFNγ+= 0.164%<br>TNFα+= 0.295%<br>IL-2+= 0.214%<br>IL-4+= 0.015%<br>IL-13+= 0.015%<br>• CD8<br>IFNγ+= 0.126%<br>TNFα+= 0.087%<br>IL-2+= 0.056%		
**Tebas, INO-4800**<br>**(40)**<br>**High Dose**	SARS-CoV-2	Human	Yes- Week 6	• CD4+<br>IFNγ+, TNFα+, IL-2+, IL-4+,<br>CD45RA+, CCR7- (Effector)<br>CD45RA-, CCR7- (Effector memory)<br>CD45RA-, CCR7+ (Central Memory)<br>• CD8+<br>IFNγ+, TNFα+, IL-2+, IL-4+,<br>CD45RA+, CCR7- (Effector)<br>CD45RA-, CCR7- (Effector memory)<br>• CD45RA-, CCR7+ (Central Memory)	• CD4<br>TNFα+: 0.02%<br>• CD8<br>IFNγ/IL-2/TNFα+: 0.11%	Overlapping Spike GP peptide pools.	• Largest responses in the high dose group.<br>• No significant Th2 responses.<br>• High CD8+polyfunctionality responses recorded.<br>• CD8+ responses were balanced among effector, effector memory and central memory, while the CD4+ population were mostly central memory.<br>• In the low dose group, CD8+ response were IFNγ+ monofunctional.<br>• CD4+ response were polyfunctional (IFNγ+ /IL-2+/TNFα+) in both dose groups.
**Sadoff, 2021 (23)**<br>**Low dose**	SARS-CoV-2 Ad26.COV.2.S	Human	Yes- Week 2	• CD4+<br>IFNγ+, IL-2+, IL-4+, IL-5+, IL-13+, CD40L+<br>• CD8+<br>• IFNγ+ and IL-2+	• CD4<br>IFNγ+/IL-2+: 0.08%<br>IFNγ+/IL-2+: 0.09% (≥65yo cohort)<br>• CD8<br>IFNγ+ and IL-2+: 0.07%<br>IFNγ+ and IL-2+: 0.06% (≥65yo cohort)	Overlapping Spike GP peptide pools.	• Polyfunctionality was skewed towards Th1 responses.<br>• Two participants in this trial recorded a measurable Th2 response.<br>• Lower CD8+ responses were found in older adults than younger adults.
**Sadoff, 2021 (18)**<br>**High dose**				• CD4+<br>IFNγ+, IL-2+, IL-4+, IL-5+, IL-13+, CD40L+<br>• CD8+<br>• IFNγ+ and IL-2+	• CD4<br>IFNγ/IL-2+: 0.11%<br>IFNγ/IL-2+: 0.11% (≥65yo cohort)<br>• CD8<br>IFNγ+ and IL-2+: 0.09%<br>IFNγ+ and IL-2+: 0.02% (≥65yo cohort)		
**Logunov, 2020**<br>**(16)**	SARS-CoV-2 AdH26-AdH5-Vac	Human	NA	• CD4+ and CD8+	• CD4+ = 2.5%<br>• CD8+ = 1.3%	NA	• Higher CD4+ and CD8+ responses in the frozen

	SARS-CoV-2 AdH26-AdH5-Lyo			• CD4+ and CD8+	• CD4+ = 1.3%<br>• CD8+ = 1.1%		formulation compared to the lyophilised formulation
**Keech, 2020 (39)**<br>**All dosage groups**	SARS-CoV-2 NVX-CoV2373	Human	Yes- Week 4 Adjuvanted Vaccine groups	• CD4+<br>IFNγ, TNFα, IL-2, IL-5, IL-13<br>• CD8+<br>IFNγ+, TNFα+, IL-2+	• NA	NA	• Strong Th1 responses<br>• Minimal Th2 responses<br>• Adjuvanted groups induced highest polyfunctional CD4+ responses
**Sahin, 2020 (30)**<br>**All dosage groups**	SARS-CoV-2 BNT162b1	Human	Yes – D29	• CD4+<br>IFNγ+, IL-2+, IL-4+, IL-13+<br>• CD8+<br>IFNγ+, IL-2+	• NA	Overlapping peptide pool of vaccine encoded full length RBD	• Strong functional and proinflammatory Th1 responses induced<br>• Minimal Th2 responses
**Xia, 2020 (38)**	SARS-CoV-2 Inactivated	Human	No	• CD4+<br>IFNγ+, IL-2+, IL-4+, IL-5+, IL-10+, Il-12+, IL-13+, TNFα+, IL-17+, IL-21+	• NA	• NA	• No changes in T cell population subset functionality
**Jackson, 2020* (24)**<br>**Low Dose**	SARS-CoV-2 mRNA-1273	Human	Yes – D43 (W6)	• CD4+CD3+<br>IFNγ+, TNFα+, IL-2+, IL-4+, IL-13+<br>• CD8+CD3+<br>IFNγ+, TNFα+, IL-2+	**<u>S1 peptide pool</u>**<br>• CD4+ Th1 response = 0.09%<br>• CD4+ Th2 response = **<br>**<u>S2 peptide pool</u>**<br>• CD4+ Th1 response = 0.2%<br>• CD4+ Th2 response = **	Overlapping peptide pool of Spike 1 and 2 peptides (S1/2 separate analysis)	• Low and intermediate-dose elicited CD4+ responses.<br>• Th1 bias observed.<br>• Effector function hierarchy: TNFα>IL-2>IFNγ<br>• Boost required for any notable polyfunctionality<br>• Low CD8 responses: responses achieved only after boost injection<br>• Minimal Th2 responses overall
**Jackson, 2020* (24)**<br>**Intermediate dose**					**<u>S1 peptide pool</u>**<br>• CD4+ Th1 response = 0.1%<br>• CD4+ Th2 response = **<br>**<u>S2 peptide pool</u>**<br>• CD4+ Th1 response = 0.22%<br>• CD4+ Th2 response = **		
**Koch, 2020 (42)**<br>**Low Dose**	MERS-CoV MVA	Human	NA<br>IFNγ only assessed	• CD4+<br>CD3+CD4+IFNγ+<br>• CD8<br>CD3+CD8+IFNγ+	• NA	Overlapping Spike GP peptide pools	• Higher responses in the low dose group compared to the high dose group
**Modjarrad, 2019 *(45)**<br>**All Doses (Low, Inter, High)**	MERS-COV DNA	Human	Yes - D98	• CD4+CD3+<br>IFNγ+, TNFα+, IL-2+, IL-4+,<br>IFNγ+ TNFα+,<br>IFNγ+ and/or TNFα+,<br>IFNγ+ and/or TNFα+ and/or IL-2<br>• CD8+CD3+<br>IFNγ+, TNFα+, IL-2+, IL-4+,<br>IFNγ+ TNFα+,<br>IFNγ+ and/or TNFα+,<br>IFNγ+ and/or TNFα+ and/or IL-2	• CD4<br>IFNγ+ = 0.14%<br>TNFα+ = 0.05%<br>IL-2+ = 0.04<br>IL-4+ = 0.025%<br>IFNγ+ TNFα+ = 0.03%<br>IFNγ+ and/or TNFα+ = 0.175%<br>IFNγ+ and/or TNFα+ and/or IL-2 = 0.18%<br>• CD8<br>IFNγ+ = 0.17%<br>TNFα+ = 0.1%<br>IL-2+ = 0.2%<br>IL-4+ = 0.045%<br>IFNγ+ TNFα+ = 0.075%<br>IFNγ+ and/or TNFα+ = 0.19%<br>IFNγ+ and/or TNFα+ and/or IL-2 = 0.27%	Overlapping peptide pool of entire spike GP	• CD4+ and CD8+ polyfunctionality recorded 2 weeks following 3rd vaccination
**Martin, 2008 (43)***<br>**Experimental**	SARS-CoV-1 DNA	Human	NA	• CD4+CD3+<br>IFNγ+, IL-2+<br>• CD8+CD3+<br>IFNγ+, IL-2+	• CD4<br>IFNγ+ =0.11%<br>• CD8<br>IFNγ+ = -	Vaccine insert specific peptide pools	• CD4+ responses higher in magnitude and frequency than CD8+ response<br>• CD8+ only registered in 2 study subjects
**Yu, 2020 (54)**<br>**All groups**	SARS-CoV-2 DNA	NHP	Yes – D3-5 (W5)	• CD4+CD3+<br>IFNγ+, IL-4+<br>• CD8+CD3+<br>IFNγ+, IL-4+	• NA	Overlapping peptide pool of Spike 1 and 2 peptides	• CD8/4+IFNγ responses stimulated<br>• CD4/8+ IL-4 responses minimal<br>• S1/RBD groups recorded the lowest responses.

							• Results suggestive of a Th1 bias response
**Muthumani, 2015*(56)<br>Low dose**	MERS-CoV DNA	NHP	Yes – D70 (W10)	• CD3+CD4+<br>IFNγ+, TNFα+, IL-2+<br>(Memory)<br>• CD3+CD8+<br>IFNγ+, TNFα+, IL-2+<br>(Memory)	• CD3+CD4+<br>IFNγ+ = 0.03%<br>TNFα+ = 0.15%<br>IL-2+ = 0.015%<br>• CD3+CD8+<br>IFNγ+ = 0.125%<br>TNFα+ = 0.12%<br>IL-2+ = 0.024%	6 peptide pools of entire Spike GP	• High dose group produced highest polyfunctionality, followed by low dose group<br>• Cytokine hierarchy = IFNγ>TNFα>IL-2
**Muthumani, 2015*(56)<br>High dose**					• CD3+CD4+<br>IFNγ+ = 0.11%<br>TNFα+ = 0.22%<br>IL-2+ = 0.021%<br>• CD3+CD8+<br>IFNγ+ = 0.22%<br>TNFα+ = 0.3%<br>IL-2+ = 0.025%		
**Erasmus, (2020) (55)**	SARS-CoV-2	NHP	NA	• IFNγ+, IL-2+, IL-17a+, TNFα+, MIPß, Granzyme b/CD107a	NA	NA	• A non-significant increase of IFNγ producing CD4+.CD8+ t cells.<br>• Slight increase in S=specific effector memory CD8+ T cells
**Corbett, 2020*<br>(48)<br>Low dose**	SARS-CoV-2 mRNA-1273	NHP	Yes – D56 (W8)	• CD3+CD4+<br>IFNγ+, TNFα+, IL-2+<br>(Th1 memory response)<br>CD40L<br>(B-Cell activation)<br>• CD3+CD8+<br>IL-4, IL-13<br>(Th2 memory response)<br>• IL-21<br>(Tfh central memory response)	• Th1 = 0.02%<br>• Th2 = -<br>• CD40L = 0.04%<br>• Tfh = 0.05%	Overlapping peptide pool of Spike 1, 2 and NP	• Highest Th1 response in high dose group<br>• Dose-dependent increase in Th1 responses following 2nd vaccination<br>• Minimal to undetectable Th2/CD8 responses.
**Corbett, 2020<br>(48)*<br>High dose**					• Th1 = 0.1%<br>• Th2 = -<br>• CD40L = 0.1%<br>• Tfh = 0.2%		
**Mercado,2020<br>(46)**	SARS-CoV-2 AdH26	NHP	Yes - D28 (W4)	• CD3+CD4+<br>IFNγ+, TNFα+<br>• CD3+CD8+<br>IFNγ+, TNFα+	• NA	Overlapping Spike GP peptide pools	• Higher IFNγ+ CD8 responses compared to IFNγ+CD4 responses<br>• Minimal IL-4 responses suggesting Th1 responses
**Kobinger, 2007<br>(57)<br>Experimental**	SARS-CoV-1 AdC7/H5	NHP	NA<br>IFNγ only assessed	• CD4+<br>IFNγ+<br>• CD8+<br>IFNγ+	• NA	5 peptide pools of spike GP	• CD8+IFNγ+ was the predominant cellular response

SARS-CoV-2: Severe acute respiratory syndrome coronavirus 2, MERS-CoV: Middle east respiratory syndrome, SARS-CoV-1: Severe acute respiratory syndrome coronavirus 1, AdH5: Recombinant human adenovirus 5, CD3/4/8: Cluster of differentiation 3/4/5, TNFα: Tumour necrosis factor alpha, IFNγ: Interferon gamma, IL-2/4/13: Interleukin-2/4/13, GP: Glycoprotein, MVA: Recombinant modified Vaccina virus Ankara, DNA: Deoxyribonucleic acid, S1: Spike domain 1, S2: Spike domain 2, Th1: T helper type 1, Th2, T helper type 2, mRNA: messenger ribonucleic acid, NHP: Non-human primate, AdC7: Recombinant chimpanzee adenovirus-7, CD40L: Cluster of differentiation 40 ligand, Tfh: T follicular helper cell, AdH26: Recombinant human adenovirus-26, NP: Nucleoprotein
